# Eukaryotic-like microtubules and dynamic instability of Asgard archaeal tubulins

**DOI:** 10.1126/sciadv.aeh1082

**Published:** 2026-07-23

**Authors:** Jan Löwe, Andriko von Kügelgen, Vicente J. Planelles-Herrero, Martin B. L. McAndrew, María A. Oliva, Julian Vosseberg, Stephan Köstlbacher, Jennah E. Dharamshi, Kathryn E. Appler, Fraser I. MacLeod, Stephanie-Jane Nobs, Steffen L. Jørgensen, Brendan P. Burns, Brett J. Baker, Tanmay A. M. Bharat, Emmanuel Derivery, Daniel Tamarit, Thijs J. G. Ettema

**Affiliations:** ^1^ MRC Laboratory of Molecular Biology, Cambridge CB2 0QH, UK.; ^2^ Centro de Investigaciones Biológicas Margarita Salas - Consejo Superior de Investigaciones Científicas, Madrid 28040, Spain.; ^3^ Laboratory of Microbiology, Wageningen University & Research, Wageningen, Netherlands.; ^4^ Department of Cell and Molecular Biology, Science for Life Laboratory, Uppsala University, Uppsala, Sweden.; ^5^ Department of Marine Science, University of Texas at Austin, Marine Science Institute, Port Aransas, TX 78373, USA.; ^6^ School of Biotechnology and Biomolecular Sciences, University of New South Wales, Sydney, NSW 2052, Australia.; ^7^ Centre for Deep Sea Research, Department of Earth Science, University of Bergen, Bergen N-5020, Norway.; ^8^ Department of Integrative Biology, University of Texas at Austin, Austin, TX 78701, USA.; ^9^ Theoretical Biology and Bioinformatics, Department of Biology, Faculty of Science, Utrecht University, Utrecht, Netherlands.

## Abstract

Eukaryotic cells change their shapes, actively segregate their DNA, and contain membrane networks, facilitated by a complex cytoskeleton containing actin filaments, microtubules made from tubulin, and other components. These filaments have ancient evolutionary origins because actin- and tubulin-like proteins form prokaryotic cytoskeletons in archaea and bacteria. Bona fide eukaryotic F-actin can be traced back to crenarchaea and Asgard archaea, which are the closest known relatives of eukaryotes. A possible Asgard archaeal origin of microtubules was suggested recently with the discovery of a lokiarchaeon containing AtubAB mini microtubules that share architectural features with their eukaryotic counterparts. Using phylogenetic analyses of metagenomic data, here we report the broad occurrence of tubulins in Asgard archaea. Biochemical and structural analyses showed that one of our previously unidentified heimdallarchaeial AtubAB tubulin pairs forms four-protofilament mini microtubules that show dynamic instability and are inhibited by the tubulin drug maytansine. Our work raises the possibility that microtubule architecture and dynamics evolved in Asgard archaea prior to eukaryogenesis.

## Teaser

Asgard archaeal mini microtubules suggest an archaeal origin of eukaryotic microtubules.

## INTRODUCTION

The eukaryotic cytoskeleton is one of the defining features of cellular complexity, enabling precise spatial organization, cell division, directed transport, motility, and the control of cell shape. It was shown previously that the two main filament-forming proteins of the eukaryotic cytoskeleton, actin and tubulin, have ancient prokaryotic (bacterial and archaeal) homologs, whose often dynamic filaments perform similar principal functions and have similar but not identical structures (*1*, *2*). Here, we studied the evolutionary history of one key component of the eukaryotic cytoskeleton, microtubules, which are made of tubulins.

The fundamental unit of microtubules consists of α- and β-tubulin heterodimers. These tubulins are closely related in amino acid sequence, each binding a GTP (guanosine triphosphate) nucleotide. The GTP bound to α-tubulin, trapped in the middle of the heterodimer, is not hydrolyzed because of lysine 254 (K254; pig tubulin numbering) in the T7 loop of β that protrudes into the GTP-binding site of α. The corresponding T7 loop of α that protrudes into the GTPase site of β has the catalytic E254 in the same position and facilitates polymerization-dependent guanosine triphosphatase (GTPase) switching by completing the active site (*3*).

Heterodimers polymerize into alternating (… ABAB …) polar protofilaments (pfs) (*4*). In most eukaryotic microtubules, 13 pfs associate laterally to form a hollow cylindrical tube with a diameter of ∼25 nm. Overall, 13-pf microtubules are a three-start pseudohelix, with 12 times α-α and β-β lateral interactions and one seam that has α-β and β-α contacts (B and A lattices, respectively) (*5*).

Microtubules can be highly dynamic, elongating, and disassembling through the addition and loss of αβ-tubulin heterodimers, a behavior energetically driven by GTP binding, intrinsic GTPase activity upon polymerization, and guanosine diphosphate (GDP) to GTP exchange. Polymerization triggers a conformational change, termed the cytomotive switch (*6*), in both α- and β-tubulins, which also leads to a reorganization of the α-β longitudinal interface. The cytomotive switch makes it possible for microtubule ends (and even single protofilaments) to have different rates of the addition of subunits, called the plus and minus ends.

Microtubules are most spectacularly used in the mitotic spindle of all eukaryotes, where their dynamics facilitate the search for chromosome attachments and their depolymerization drives chromosome segregation. One hallmark of microtubule dynamics is dynamic instability (*7*) that describes when heterodimer addition to the plus end is slower than GTP hydrolysis. This means that the structure is no longer capped with stabilizing GTP-bound tubulins and catastrophically depolymerizes almost instantaneously, due to the strain the GDP state causes inside the microtubule.

Beyond α- and β-tubulins, additional members of the tubulin superfamily such as γ, δ, ε, and cryptic carry out specific functions, including microtubule nucleation, basal body organization, and the fine-tuning of microtubule architecture across diverse cellular contexts (*8*). The limited sequence similarity between eukaryotic tubulins and their known prokaryotic counterparts has long obscured the evolutionary origins of microtubules. Tubulin-like proteins (distant homologs of tubulin) in bacteria, archaea, and viruses, such as FtsZ, CetZ, TubZ, PhuZ, and artubulins (*9*), bear structural similarity to eukaryotic tubulins, form similar protofilaments but display only limited sequence similarity, and are typically separated by long branches in phylogenetic trees (*8*). They have not been reported to form microtubules.

In the early 2000s, members of the genus *Prosthecobacter* within the *Verrucomicrobia* were found to contain more closely related tubulin homologs, BtubA and BtubB (*10*), which form “mini microtubules” with similar properties to eukaryotic microtubules but with greatly reduced protofilament numbers of four to five (*11*, *12*). BtubAB bacterial tubulins, of which there are very few known sequences, cluster within the eukaryotic tubulin clade in phylogenetic trees (*8*), implying that they may have been acquired by horizontal gene transfer (HGT).

Research into the evolution of eukaryotes and their cytoskeleton, has recently been invigorated by the discovery of Asgard archaea [also known as Asgardarchaeota (*13*) or Prometheoarchaeota (*14*)], the closest known archaeal relatives of eukaryotes (*15*–*17*). Asgard archaeal genomes encode a diverse molecular toolkit of eukaryotic signature proteins (*18*, *19*), with rapidly accumulating evidence indicating structural and functional conservation that predates eukaryogenesis—for example, for actin (*20*), endosomal sorting complexes required for transport (*21*), and soluble *N*-ethylmaleimide–sensitive factor attachment (SNARE) proteins (*22*).

While close actin homologs can be found in most Asgard archaeal genomes (and crenactin in some Crenarchaeota, now classified as Thermoprotei) (*23*), close tubulin homologs have only been found in Odinarchaeia (*17*) and in two related Lokiarchaeia species (*24*, *25*). Both odinarchaeial and lokiarchaeial tubulins have been shown to form eukaryotic-like protofilaments (*25*, *26*), with the lokiarchaeial tubulins AtubAB forming five-pf mini microtubules (*25*). However, the unclear distribution of tubulin genes across Asgard archaeal genomes and the scarcity of detailed structural and functional studies currently limit our ability to infer their functional roles and to draw robust conclusions about the evolutionary origins of the eukaryotic microtubule cytoskeleton.

Here, we expand the set of known prokaryotic tubulins, with the aim of clarifying their evolutionary origin. We report the presence of close tubulin homologs in several previously unidentified Asgard archaeal metagenome-assembled genomes (MAGs), including in the closest Asgard archaeal relatives of eukaryotes. Phylogenetic analyses of these Asgard archaeal tubulin homologs revealed multiple tubulin paralogous copies clustering within the eukaryotic tubulin tree, revealing a complex evolutionary scenario of duplications and differential losses. Subsequent in-depth structural characterization of Kariarchaeaceae (Kari; Heimdallarchaeia) AtubAB tubulin homologs revealed that their mini microtubules share many key characteristics of eukaryotic microtubules, including stable heterodimers, a seam, M-loops, maytansine (MAY) inhibitor sensitivity, and dynamic instability—but differ uniquely in forming four-pf microtubules.

## RESULTS

**Fig. 1.**
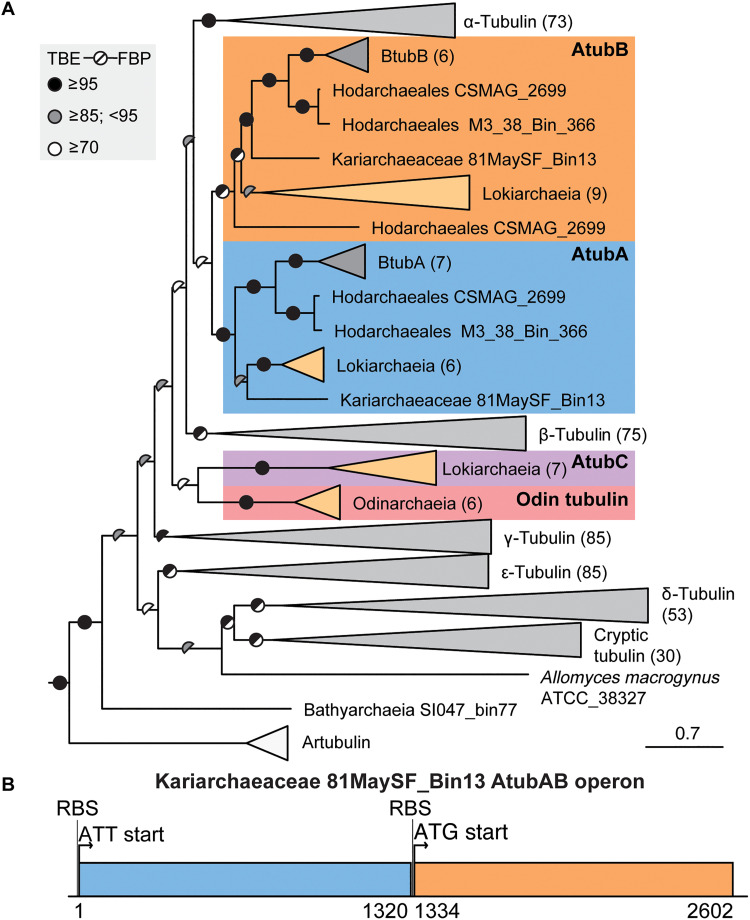
Phylogeny of tubulins reveals Asgard archaeal AtubAB tubulin genes. (**A**) Colors for collapsed clades indicate taxonomy: Asgard archaea (orange), eukaryotes (gray), bacteria (dark gray), and other archaea (white). Branch support values represent transfer bootstrap expectation (TBE) and standard Felsenstein’s bootstrap support (FBS). Phylogeny reconstructed using IQ-TREE 3 under the model Q.pfam+C50+G4+PMSF. The outgroup was removed for visualization. Branch length scale indicates substitutions per site. The full tree is shown in fig. S1A; the gene maps are shown in fig. S1B. The alignment (trimmed off sites with more than 90% gaps and sequences with more than 50% gaps) contains 477 sequences and 578 columns. (**B**) Schematic of the tubulin-like Kariarchaeaceae 81MaySF_Bin13 AtubAB operon, coding for the Kari AtubAB proteins used in this study (see also fig. S1, C and D). Arrows indicate start codons.

To establish the relationship between previously unidentified prokaryotic sequences and eukaryotic tubulins, we performed sequence homology searches and phylogenetic analyses (Materials and Methods). These analyses revealed bona fide tubulin sequences in multiple prokaryotic groups (Fig. 1A and fig. S1A). The odinarchaeial tubulins (hereafter “Odin tubulins”) cluster as a monophyletic group, formed by proteins encoded in a single copy in all known odinarchaeial genomes, indicative of a conserved function ancestral to this group. We identified a single Bathyarchaeia tubulin sequence clustered through a long branch, indicative of an ancient HGT, but contamination of this sequence could not be ruled out. Previously unidentified *Verrucomicrobia* sequences formed monophyletic groups with the BtubA and BtubB clades, expanding the known sequence diversity for these bacterial tubulins that were previously shown to form four- or five-pf mini microtubules (*11*, *12*).

Additional tubulin copies were found in Loki- and Heimdallarchaeia. One set of these, encoded in the genome of *Candidatus* Lokiarchaeum ossiferum, has been shown recently to form mini microtubules (*25*) that recapitulate many structural features of eukaryotic 13-pf microtubules, but not protofilament number. We found multiple additional tubulin homologs in Lokiarchaeia, including MAGs recovered from marine sediments sampled in the Arctic ocean, the Gulf of Mexico, and the Australian Shark Bay. In addition, three heimdallarchaeial MAGs were also found to contain tubulins. The lokiarchaeial and heimdallarchaeial tubulins formed three different monophyletic groups, which we named AtubA, AtubB, and AtubC (Fig. 1A). AtubA and AtubB were found to also contain BtubA and BtubB, respectively, which clustered with the heimdallarchaeial tubulins with high confidence, indicating an Asgard archaeal origin of the verrucomicrobial tubulins. As bacterial tubulins were previously thought to have evolved from eukaryotic tubulins (*27*), these results shed light on a long-standing debate in tubulin evolution. The AtubC clade comprises tubulin sequences from Lokiarchaeia.

AtubA, AtubB, and AtubC cluster confidently with the eukaryotic tubulin clade in most phylogenetic reconstructions (see Supplementary Discussion). In those, AtubA and AtubB mostly cluster as a sister group to α-tubulin. The AtubC clade has a more uncertain position yet is placed at the base of the α- and β-tubulin clade in most analyses, albeit with low support. Odin tubulins tend to cluster together with AtubC, indicating that they may be orthologous to the lokiarchaeial AtubC sequences (Supplementary Discussion). The position of artubulins, linked through a very long branch to the Asgard archaeal-eukaryotic tubulin clade, is never fully resolved, with main placements being at the base of the eukaryotic tubulin group or within, possibly as sister to Odin tubulins (Supplementary Discussion).

We found AtubA and AtubB to always be encoded by genes located in tandem, and AtubC is encoded within 10 kb of these genes in Lokiarchaeia (fig. S1B). A second AtubB homolog (AtubB2) is found in some lokiarchaeial genomes, at a distant position. The genomic context of these genes seems not to be conserved between taxonomic groups.

To further our understanding of their evolutionary and functional relationships, we set out to investigate the structure and dynamics of the previously unidentified Asgard archaeal Atub tubulins. One such AtubAB pair turned out to be highly accessible biochemically: Kariarchaeaceae 81MaySF_Bin13 AtubAB, hereafter AtubAB (Fig. 1B and fig. S1C). AtubA and AtubB are encoded in an operon, with the second ribosome binding site (RBS) for *atubB* partly overlapping with the stop codon of *atubA* (Fig. 1B). AlphaFold 3 predictions (*28*) revealed AtubAB to likely have the tubulin fold, form heterodimers, and form alternating (ABAB) protofilaments (fig. S1D). Inspection of the T7 loops (*3*) in the predicted protofilaments also revealed that it is likely that the GTPase site between AtubA at the top and AtubB at the bottom in the dimer is not active because of AtubA K248 not supporting hydrolysis in the GTP-binding pocket of AtubB below. In our hands, AlphaFold 3 could not predict the correct filament structure of AtubAB.

**Fig. 2.**
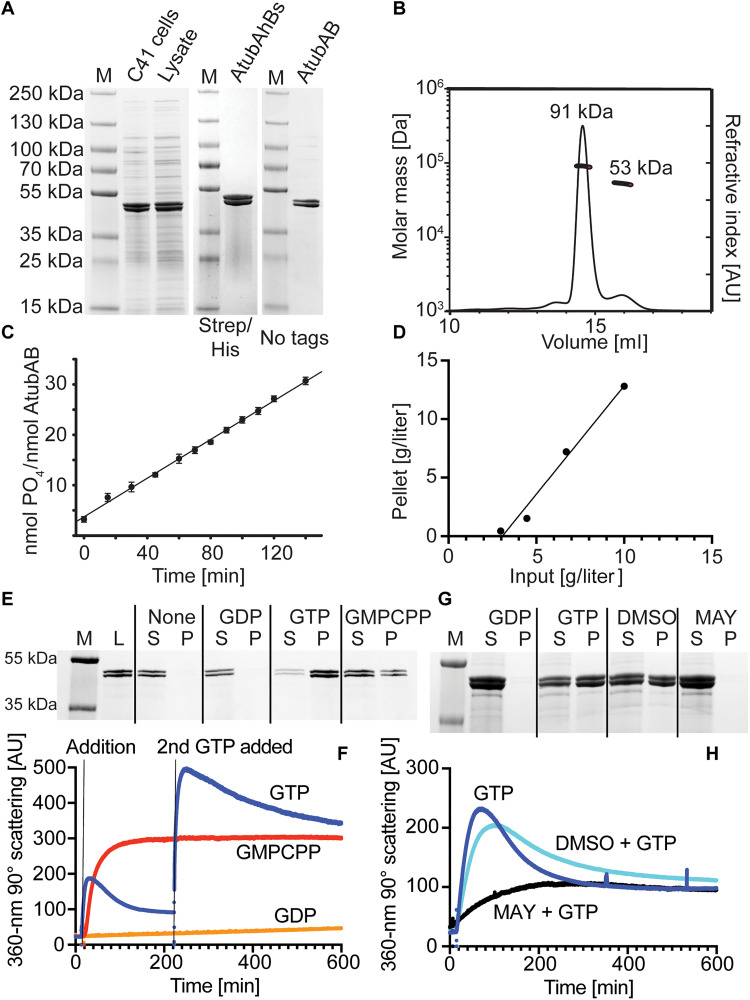
AtubAB expressed in *E. coli* forms stable heterodimers and polymerizes in the presence of GTP, which is inhibited by the tubulin inhibitor maytansine. (**A**) Coomassie-stained SDS–polyacrylamide gel electrophoresis (SDS-PAGE) gels showing strong soluble untagged AtubAB expression in *E. coli* C41(DE3) cells (left), purified His_8_ and Strep-tagged AtubAhBs (middle), and purified untagged AtubAB (right), as used in this study. (**B**) SEC-MALS reveals that purified untagged AtubAB is a stable heterodimer. AU, arbitrary units. (**C**) AtubAB is a slow GTPase: 0.192 ± 0.004 nmol phosphates released/nmol AtubAB per minute (25°C); *n* = 3. (**D**) Steady-state concentration of AtubAB polymerization in BRB80 + 500 mM potassium glutamate polymerization buffer (used throughout this study) is ∼3 g/liter (62 μM dimer). (**E**) Pelleting assay revealing that AtubAB polymerization is GTP dependent and can also be triggered by the nonhydrolyzable GTP analog guanylyl 5′-α,β-methylenediphosphonate (GMPCPP). L, loaded; S, supernatant; P, pellet. (**F**) Ninety-degree 360-nm light scattering assay showing the same GTP/GMPCPP dependence of AtubAB polymerization as in (E). GTP hydrolysis leads to depolymerization; adding GTP again leads to repolymerization. (**G**) Pelleting assay showing the inhibition of GTP-induced AtubAB polymerization by the tubulin inhibitor MAY. Dimethyl sulfoxide (DMSO) and MAY reactions also contained GTP (see also fig. S2D). (**H**) Ninety-degree 360-nm light scattering assay showing the same maytansine inhibition of GTP-induced AtubAB polymerization as in (G).

For bacterial expression of AtubAB, we constructed two bicistronic expression vectors, pHis17-AtubAB for untagged expression and pHis17-AtubAhBs for His_8_(h)- and Strep(s)-tagged AtubAB, respectively (Materials and Methods and fig. S2, A and B). Small-scale expression tests revealed that AtubAB expresses highly in *Escherichia coli* C41(DE3) cells, making up a large proportion of the soluble protein content of the cells (Fig. 2A, left). Nickel-based purification of AtubAhBs pulled out AtubAh and AtubBs simultaneously (Fig. 2A, middle), although only AtubAh had a His_8_ tag, indicating that AtubAB forms a stable heterodimer. Large amounts of protein (>100 mg) could be purified, which led us to attempt purifying untagged AtubAB by anion exchange chromatography, followed by size exclusion chromatography (SEC; Materials and Methods). Again, large amounts of protein could be purified (>100 mg), and AtubAB cofractionated throughout the procedure despite not adding GTP to the buffers and using high salt conditions during the anion exchange chromatography (Fig. 2A, right). SEC coupled with multiangle light scattering (SEC-MALS) of the purified untagged AtubAB sample revealed a single peak with an estimated mass of 91 kDa, close to the expected mass of 94 kDa of the AtubAB heterodimer (Fig. 2B). AtubAB is a slow GTPase under the conditions used: 0.192 ± 0.004 nmol phosphates released/nmol AtubAB per minute (25°C; Fig. 2C and Materials and Methods).

Attempts to polymerize AtubAB revealed that standard tubulin buffers such as BRB80 (Materials and Methods) did not yield filaments as investigated by pelleting and negative staining electron microscopy (EM). After screening other conditions, we settled on the inclusion of 500 mM potassium glutamate in BRB80 as the polymerization buffer used throughout this study. This has been used earlier with eukaryotic tubulin (*29*) and was also found to polymerize Lokiarchaeial AtubAB best (*25*). The steady-state concentration of untagged AtubAB in polymerization buffer in the presence of GTP was determined by a pelleting assay to be ∼3 g/liter (32 μM dimer) (Fig. 2D). Pelleting revealed that the polymerization of AtubAB is strictly dependent on GTP or its nonhydrolyzable analog, GMPCPP (guanylyl 5′-α,β-methylenediphosphonate; Fig. 2E). It also showed that AtubAB pellet together, stoichiometrically. Ninety-degree 360-nm light scattering was used as an alternative method to confirm the nucleotide dependence of polymerization of AtubAB and to show GTP hydrolysis–driven depolymerization, which was absent in the GMPCPP-polymerized sample (Fig. 2F). When we analyzed the dynamics of tagged AtubAhBs protein in the same light scattering assay, we found that the tag altered its behavior, leading to enhanced polymerization and faster depolymerization (fig. S2C).

Because the AtubAB proteins are closely related to eukaryotic tubulins in sequence (∼35 to 43% sequence identity, depending on the tubulins used), we then tested an array of tubulin-directed drugs and compounds that either stabilize or disrupt microtubule formation (fig. S2D). MAY, a small-molecule compound preventing microtubule polymerization by binding and sequestering tubulin dimers, showed strong inhibition of AtubAB polymerization, both in pelleting and in the light scattering assay (Fig. 2, G and H). A binding assay with a fluorescein-maytansinoid (FcMaytansine) revealed a dissociation constant (*K*_d_) of 87 ± 17 nM (mean ± SEM, *n* = 4; see Materials and Methods and fig. S2Ei), to be compared with 6.8 nM for FcMaytansine:tubulin (*30*). A subsequent fluorescence anisotropy competition experiment with FcMaytansine and maytansine revealed a *K*_d_ value for (unlabeled) maytansine against AtubAB of 5.1 ± 0.2 μM. This is in the same range as maytansine binding to eukaryotic tubulin [∼900 nM (*31*)] (fig. S2Eii). A superposition of the AtubAB AlphaFold 3 model with a structure of maytansine bound to β-tubulin [Protein Data Bank (PDB) 4TV8] (*32*) revealed that the maytansine binding site is highly conserved (fig. S2F). We conclude that AtubAB is similar to eukaryotic αβ-tubulins in terms of its biochemical properties, polymerization behavior, and its susceptibility to the inhibitor maytansine.

**Fig. 3.**
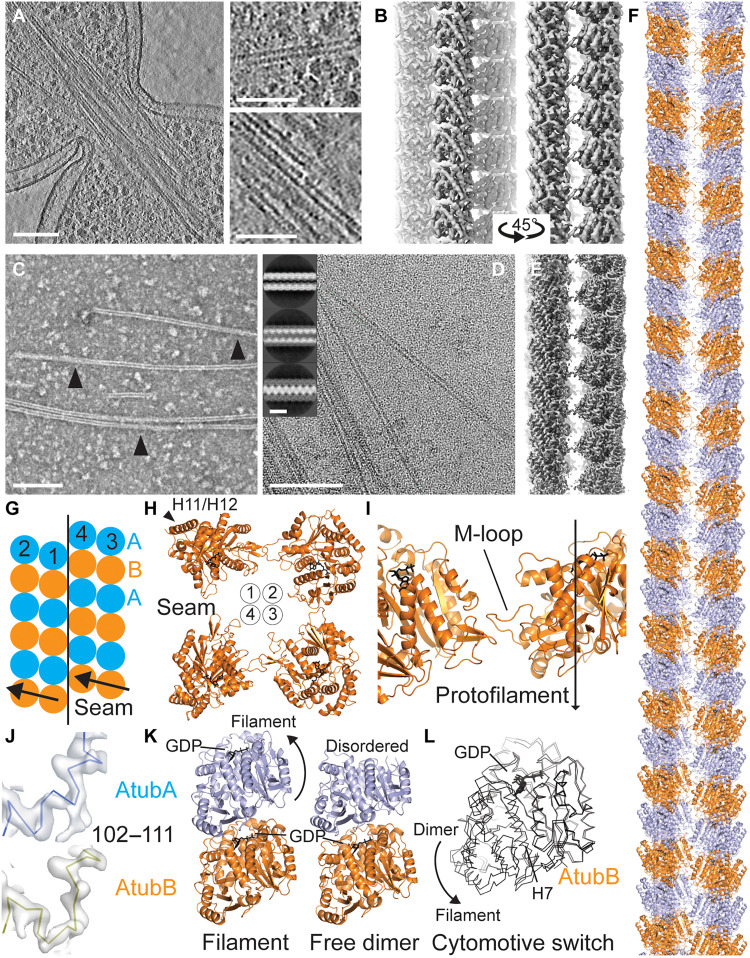
AtubAB cryo-EM mini microtubule structures in cell in *E. coli* (overexpression) and in vitro after purification. (**A**) Electron cryotomogram of a FIB-milled lamella of an *E. coli* C41(DE3) cell overexpressing untagged AtubAB, revealing filaments passing a cell division site (left overview, right magnified). See movie S1. Scale bars, 100 nm (left) and 50 nm (right). (**B**) Model-free helical subtomogram average (fig. S3B), calculated from tomograms as in (A), revealing a four-stranded mini microtubule at 6.4-Å resolution. (**C**) Negative staining electron micrograph of untagged AtubAB polymerized in BRB80 + 500 mM potassium glutamate polymerization buffer, revealing filaments as seen in (A) and (B) (scale bar, 100 nm). Arrowheads mark crossovers, indicating twisting filaments. (**D**) Cryo-EM micrographs of AtubAB polymers in polymerization buffer (scale bar, 100 nm); insets: two-dimensional (2D) classes (scale bar, 10 nm). (**E**) Cryo-EM structure of untagged AtubAB filaments after helical single particle processing, at a resolution of 3.1 Å, revealing the same four-stranded mini microtubules as in (B) (fig. S3E). (**F**) Fitted atomic model of the AtubAB four-stranded mini microtubule. AtubA: blue; AtubB: orange. See movies S2 and S3. (**G**) Schematic of the pseudohelical symmetry of AtubAB mini microtubules, leading to a seam. (**H**) Top view of the four mini microtubule protofilaments. Helices H11 and H12 of the tubulin fold point outward. (**I**) AtubAB mini microtubule protofilaments are held together by M-loops (residues ∼270 to 280). See movie S3. (**J**) Cryo-EM density regions demonstrating the identification of AtubA and AtubB after symmetry expansion and 3D classification. (**K**) Comparison of the AtubAB heterodimer in the mini microtubule filament [as shown in (F); left] and the structure of unpolymerized, untagged AtubAB as determined by single particle cryo-EM (right). (**L**) Superimposing the AtubBs in (K) reveals the polymerization-dependent cytomotive switch in AtubAB. See movie S4.

To determine the structure of AtubAB was challenging because the organism from which the AtubAB metagenomic sequences came originally has not been cultivated or isolated. Therefore, we first determined the in-cell structure of AtubAB after overexpression in *E. coli* C41(DE3) cells as described above (Fig. 2A, left). The cells were thinned by focused ion beam (FIB) milling into lamellae <200 nm thick (fig. S3A). Subsequent cryo–electron tomography (cryo-ET) of the lamellae revealed bundles of filaments filling large areas of the *E. coli* cells in a way that could interfere with cell division (Fig. 3A and movie S1). Some single filaments were identified. These mostly appeared as two lines of density along the filament axis (Fig. 3A, right). Model-free subtomogram averaging (STA) of the filaments using helical symmetry as implemented in Relion 5 (*33*) (Materials and Methods and fig. S3B) resulted in a 6.4-Å resolution map that revealed a four-stranded (four-pf), slightly twisting mini microtubule structure (Fig. 3B). Symmetry parameters were twist = −89.9° and rise = 10.3 Å.

To reveal further molecular details of the AtubAB mini microtubules, we turned to EM of purified material, using untagged AtubAB protein in the BRB80 polymerization buffer containing 500 mM potassium glutamate. Negative staining EM revealed slowly twisting filaments with two parallel lines and occasional crossovers (Fig. 3C). Filament formation was again found to be strictly dependent on the presence of GTP or GMPCPP (fig. S3C). In cryo-EM, the filaments were often bundled, so manual picking was used to pick single filaments (Fig. 3D). Subsequent particle extraction and two-dimensional (2D) classification revealed classes that are consistent with a four-pf structure (Fig. 3D, insets). Helical reconstruction of the cryo-EM data produced a map at 3.1-Å resolution that was very similar to the cryo-ET in-cell structure but at higher resolution. The reconstruction used helical symmetry with a twist of −89.84° and a rise of 10.46 Å (Fig. 3E and fig. S3, D and E). Because AtubAB formed stable heterodimers even outside of the filaments (Fig. 2B) and pelleted filaments contained both proteins (Fig. 2E), we assumed that the structure consists of ABAB alternating protofilaments (Fig. 3F). As a consequence, the helical symmetry observed means that the structure must be pseudohelical and contain a seam, just as in eukaryotic 13-pf microtubules (Fig. 3, G and H), in which lateral contacts change from A-to-A and B-to-B to A-to-B and B-to-A (called B and A lattices, respectively, in eukaryotic microtubules). Moreover, the C-terminal helices 11 (H11) and H12 of the tubulin fold point outward in AtubAB mini microtubules (Fig. 3H), just as they do in eukaryotic microtubules.

The way protofilaments in AtubAB mini microtubules are held together laterally is highly reminiscent of eukaryotic microtubules (Fig. 3I). An M-loop (microtubule loop) (*34*), formed in AtubA and AtubB by residues ∼270 to 280, reaches over from the C-terminal domain of one subunit to the GTPase domain of another, contacting a hydrophobic pocket mainly through a single phenylalanine residue (F275 in AtubA and F278 in AtubB). The M-loop is the only interprotofilament contact holding the entire structure together through repeated contacts that increase the avidity of this interaction.

We then aimed to differentiate AtubA and AtubB in the cryo-EM map. For this, we used eightfold symmetry expansion with subsequent 3D classification, focusing on a single dimer (Materials and Methods and fig. S3D, box). The resulting map allowed AtubA and AtubB to be distinguished both visually in a specific region of the map (Fig. 3J) and, computationally, by automated model building using ModelAngelo (*35*), which produced complete atomic models for both AtubA and AtubB (fig. S3D). While our procedure did not resolve the seam position in each mini microtubule observed (for which there was not enough signal because of the 38% sequence identity between AtubA and AtubB), it was sufficient to reveal the alternating nature of each protofilament and enable us to build a complete AtubAB mini microtubule structure with a seam, as required by the symmetry (Fig. 3, F and G, and movie S3).

Comparison of the resulting structure with the cryo-ET in-cell structure revealed an excellent fit, meaning that AtubAB forms the same four-pf mini microtubules when expressed in *E. coli* and when polymerized in vitro (fig. S3E). Comparison of the AtubAB heterodimer in the filament with eukaryotic αβ-tubulin revealed very small deviations, with root mean square deviations between AtubA and β-tubulin (PDB 7QUP) of 0.9 Å and between AtubB and α-tubulin (PDB 7QUP) of 0.92 Å, emphasizing their close similarity at the subunit level (fig. S3F and movie S2). Unexpectedly, we found that the AtubAB mini microtubules only contain GDP as indicated by the map. This matched our biochemical finding that purified AtubAB contained only GDP (fig. S3G). The lack of unhydrolyzed GTP trapped between AtubA and AtubB, as is seen in tubulin between α- and β-tubulins, is likely due to the time taken for purification using anion exchange and SEC, which took a day and was performed in buffers that lack GTP (Materials and Methods). The AtubAB GDP heterodimer was still stable (Fig. 2B).

Because dynamic actin and tubulin filaments undergo a cytomotive switch (*6*) that depends on polymerization-induced changes in monomer structure, we compared monomer and polymer structures of AtubAB. Using a cryo-EM single particle analysis (SPA), we solved the structure of unpolymerized and untagged AtubAB at 3.5-Å resolution (fig. S3, H and I). Parts of the GTPase site on top of AtubA were not resolved in the map, likely because no nucleotide had been added to the sample, meaning the site might be empty and hence disordered (Fig. 3K). This is likely due to our purification protocol being unable to maintain nucleotide binding in the solvent-exposed AtubA GTP-binding site. Comparison of the AtubAB structures in the filament and unpolymerized (free) heterodimer forms revealed two major differences. First, the intersubunit angle changed. Second, each subunit underwent tubulin’s canonical polymerization-state–dependent cytomotive switch, in which the C-terminal domain, including H7 (*3*), moves downward upon polymerization (Fig. 3, K and L, and movie S4). This is the same conformation change as in all other tubulins and tubulin-like proteins (*6*). Data of the structural work are summarized in table S1.

The structural work revealed that AtubAB forms mini microtubules (movie S3) that share many features with eukaryotic microtubules, including the following: the subunit fold (movie S2), overall polarity, alternating protofilaments, polymerization-dependent GTPase activity, pseudohelical symmetry with a seam, C-terminal H11 and H12 helices on the outside, very little overall twist, M-loops holding protofilaments together, and, importantly, the polymerization-dependent cytomotive switch.

**Fig. 4.**
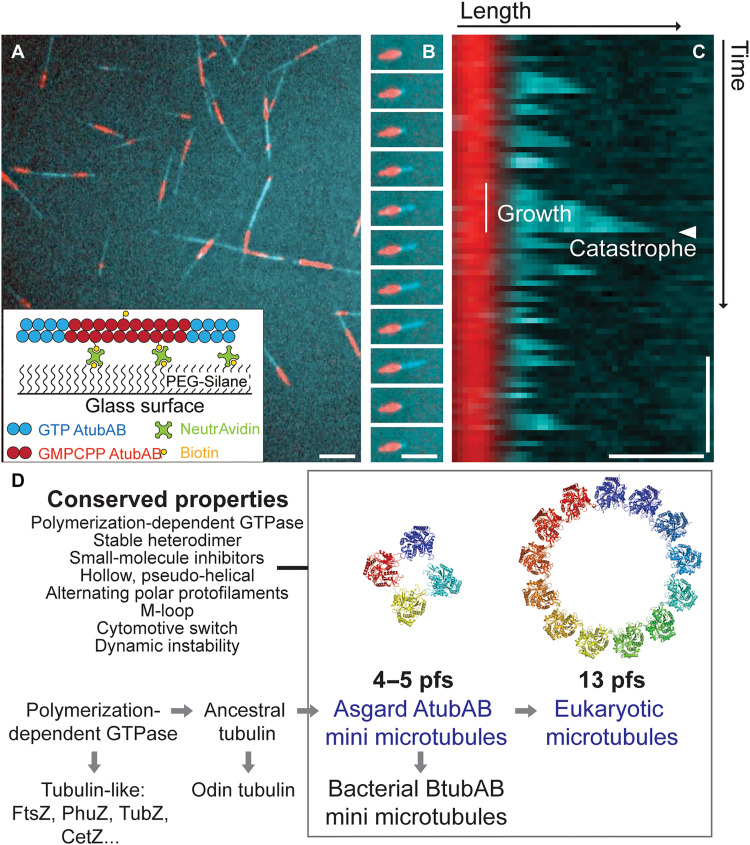
AtubAB dynamic instability revealed by TIRF microscopy. (**A**) Inset: Assay design; biotinylated, GMPCPP-stabilized AtubAB dimers (labeled with 20% Alexa Fluor 647, shown as red) were anchored via NeutrAvidin to PEG-Silane. Free AtubAB dimers (4 μM 20% Atto 488 labeled; cyan) were added, and filament polymerization was observed by TIRF microscopy. Field view revealing red GMPCPP seeds with plus- and minus-end growth (cyan). Only this used 20% tagged (unlabeled) AtubAhBs to observe dynamics at high concentrations needed for field views. Scale bar, 10 μm. (**B**) Time-lapse of plus-end growth of untagged AtubAB filament. The 10th image down shows the moment just after catastrophe, the ultrarapid depolymerization of the mini microtubule. Scale bar, 5 μm. (**C**) Kymograph (time: vertical; filament axis: horizontal) showing an untagged AtubAB filament growing and undergoing catastrophe at the plus-end. One growth episode (line) and one catastrophe event (arrowhead) are highlighted—it is the same as in panel (B). Scale bars, 2 min/2 μm. See also fig. S4 (A and B) and movie S5. (**D**) Asgard archaeal four-pf mini microtubules show all the hallmarks of eukaryotic 13-protofilament microtubules—but are much thinner. We therefore suggest that it is possible that Asgard archaeal mini microtubules are the evolutionary precursors of eukaryotic microtubules. Arrows do not indicate evolutionary distances or complexity.

Having established strong biochemical and architectural similarities of AtubAB four-pf mini microtubules with eukaryotic 13-pf microtubules, we set out to investigate filament dynamics. We labeled GMPCPP-bound stable AtubAB filament seeds with Alexa Fluor 647 and GTP-bound AtubAB with Atto 488. Some AtubAB was biotin labeled to constrain filaments to the biotin-functionalized glass surface with neutravidin (Fig. 4A and Materials and Methods). Total internal reflection fluorescence (TIRF) microscopy was then used to image the filaments, seeded by the red GMPCPP seeds, as they grew and shrank. The filaments exhibited two different growth speeds (fig. S4, A and B, and movie S5) (minus ends, median speed of growth: 0.48 μm/min; plus ends, median speed of growth: 1.80 μm/min), which is caused by their structural polarity and the kinetic difference of subunit addition to either end as caused by the cytomotive switch (*6*).

The fast growing plus end showed strong dynamic instability (Fig. 4, B and C, and fig. S4, A and B) (*7*). After a period of plus-end growth, the filaments underwent a change in behavior, leading to a process called catastrophe that involves rapid depolymerization. The polymer, which is then thought to be completely in the GDP-bound form, loses its protection through a cap of GTP-bound subunits, which allows internal strain to be released via rapid polymer disassembly. Figure S4A shows two more examples of filaments exhibiting dynamic instability at the plus end, growing to the right. They also show the slower minus-end growth (to the left), which does show dynamic instability under the conditions used. Figure S4Aiv shows the effect of adding in tagged AtubAhBs, which alters filament dynamics also in this assay (relates to light scattering in fig. S2C). Figure S4B summarizes statistics of the untagged AtubAB filament dynamics observed under the conditions used (Materials and Methods).

## DISCUSSION

Together (Fig. 4D), our analyses showed that Heimdallarchaeia AtubAB tubulins are close relatives of eukaryotic tubulins and share with them many features and behaviors. They are encoded in the genome as a partly overlapping operon with a short intergenic region (Fig. 1, A and B, and fig. S1, A to D). Expressed in *E. coli* and purified, they form stable heterodimers, bind and hydrolyze GTP, and polymerize in a crowding polymerization buffer in a strictly GTP-dependent manner. Similar to eukaryotic tubulin, AtubAB polymerization is also inhibited by the small-molecule maytansine. When expressed in bacterial *E. coli* cells, AtubAB polymerizes into four-pf mini microtubules that are hollow and pseudohelical with a seam, both in cells and after purification, in vitro. Protofilaments alternate and are held together via the conserved M-loop. The monomers undergo a polymerization-dependent cytomotive switch that enables polar dynamics with a fast plus and a slow minus end—supporting our previous supposition that this is a universal property of these polymers (*6*). Last, these polymers exhibit dynamic instability at their plus ends. Amazingly, all these properties are shared between AtubAB mini microtubules and eukaryotic microtubules, except their protofilament numbers: 4 versus 13.

What does this mean for the evolutionary relationship of AtubAB with microtubules? Let us first examine scenarios in which tubulin(s) forming microtubules were inherited vertically by early eukaryotes from their Asgard archaeal common ancestor. The presence of tubulin homologs in Asgard archaea is broad but patchy: Tubulins are only found in ∼1% of all surveyed Asgard archaeal genomes to date, in certain members of Lokiarchaeia, Odinarchaeia, and Heimdallarchaeia (Hodarchaeales and Kariarchaeaceae). High rates of gene loss are common across all domains of life, and the scenarios in which tubulin(s) forming (mini) microtubules were inherited by early eukaryotes from their Asgard archaeal precursors may therefore imply losses of multiple paralogs in most Asgard archaeal lineages, as well as in most lokiarchaeia and heimdallarchaeia. These scenarios would therefore indicate complex combinations of gene duplication and losses across the Asgard archaea. These scenarios could be supported by the coexistence of six stable paralogs in eukaryotes and by the observed elevated rates of gene duplications in Asgard archaea compared to other archaea (*15*), which may suggest that Asgard archaeal gene histories could in some cases be as complex as those observed in early eukaryotic evolution (*36*). Given the patchy distribution of these genes in Heimdallarchaeia, it is likely that there have been frequent horizontal transfer events of a subset of tubulin genes. However, the presence of tubulin homologs in a hodarchaeon and a kariarchaeon raises the possibility that a distinct set of tubulin paralogs was present in the heimdallarchaeial ancestor of eukaryotes (*15*).

An alternative, second scenario consistent with the observed data is the horizontal transfer of tubulin genes between Asgard archaeal lineages and early eukaryotic ancestors. However, there is no clear evidence of such events thus far. Currently, the lack of traces of synteny between Asgard archaeal lineages beyond the AtubAB operon or mobile elements in its vicinity prevents the identification of signals of joint transfer.

Exploring additional Asgard archaeal genomic diversity will be key to identifying AtubABC orthologs or additional paralogous genes that will help reconcile the evolution of this gene family with the Asgard archaeal species tree. In the meantime, given currently leading theories of eukaryogenesis, we suggest that microtubules evolved in Asgard archaea prior to the onset of eukaryogenesis.

Because AtubA and AtubB seem to be more closely related to α-tubulin based on phylogenetic analyses, this suggests the intriguing hypothesis that the use of a similar AB heterodimer with a GTPase defective interface between the A and B subunits arose by a process of convergent evolution. This suggests that there is something special about using a heterodimer as building block of (mini and eukaryotic) microtubules.

Our findings raise important future questions: What is the function of mini microtubules in Asgard archaea? Our discovery of maytansine as a polymerization inhibitor of AtubAB and recent advances with the cell biology of Asgard archaeal organisms (*37*) will help answering this question. Do molecular motor proteins exist in any prokaryote? This is an old question that so far has never received a positive answer. The discovery of (almost) nontwisting and stiff mini microtubules that display tubulin’s C-terminal α helices (helices H11/H12; Fig. 3H) on the outside makes it more likely than ever those archaeal motor proteins may exist and they may, or may not be, related in sequence to eukaryotic motor proteins. Their existence would also need AtubAB mini microtubule dynamics to be regulated through stabilizing (mini) microtubule-associated proteins (MAPs), whose possible existence will also need to be probed in archaea.

## MATERIALS AND METHODS

GTP lithium salt and GDP were purchased from Sigma-Aldrich, dissolved in water at 100 mM, pH adjusted with tris [saturated (sat.)] to pH 7, and aliquoted before freezing. GMPCPP was purchased from Jena Biosciences as a 10 mM solution and used as supplied. Maytansine was purchased from Merck (SML3451), dissolved at 10 mM in DMSO (dimethyl sulfoxide), and kept frozen before use. The compounds paclitaxel (Alfa Aesar Chemicals); vinblastine, colchicine, podophyllotoxin, and nocodazole (Sigma-Aldrich); epothilone A, plinabulin, and pironetin (MedChemExpress); and sabizabulin (TargetMol) were dissolved at 20 or 50 mM in DMSO and kept frozen before use. Gatorbulin was provided by H. Luesch (University of Florida), discodermolide was provided by I. Patterson (University of Cambridge), zampanolide was provided by K.-H. Altmann (ETH Zurich), and laulimalide was from R. Keyzer and was purified from sponge biomass (Victoria University of Wellington, Ministry of Fisheries, Kingdom of Toga, permit F1/40/76/16 for collecting the sponges). FcMaytansine was also provided by K.-H. Altmann (ETH Zurich).

Two different versions of the general tubulin buffer BRB80 were used. For most experiments, BRB80 was 80 mM Pipes, 1 mM MgCl_2_, and 1 mM EGTA [pH 6.9 (KOH)]. A total of 500 mM potassium glutamate was added to that, before final pH adjustment to 6.9, to produce the polymerization buffer. TIRF experiments (below) used a slightly different version of BRB80, and this did not have scientific reasons.

### Identification of tubulin homologs and phylogenetic analysis

To recover a set of tubulin sequences for phylogenetic analyses, we started by performing broad sequence similarity searches using reference datasets as query, including COG5023, COG0206, and PF00091, against ENSEMBL and National Center for Biotechnology Information (NCBI) nr databases (versions September 2022). We removed redundancy using CD-HIT (-c 0.8) (*38*), aligned these sequences using FAMSA (*39*), trimmed using trimAl (-gt 0.003) (*40*), and reconstructed a phylogeny using FastTreeMP (*41*). Using this tree, we selected sequences from an inclusive clade including all eukaryotic tubulin sequences. In addition, we used the tubulin-family sequences from (*17*) to perform a sequence similarity search using PsiBLAST (-num_iterations 1 -evalue 0.001) (*42*) against a local database including all public Asgard archaeal genomes (last updated: August 2023) and additional MAGs (published as accession numbers GCA_029880815.1 and JBSWQN000000000 or within BioProjects PRJEB8 and PRJNA504765). We combined all these sequences in a single file, removed duplicates and all sequences shorter than 100 aa, aligned again using FAMSA, trimmed out gappy columns using trimAl (-gt 0.01), and reconstructed a phylogeny using IQ-TREE v2.2.2.6 (*43*) using LG+G4. We identified long branches using PhyKIT (lbs) (*44*) and removed sequences with a score higher than 50. We then reconstructed one more tree by following the previous procedure: FAMSA, trimAl (-gt 0.01), and IQ-TREE under the model LG+G4. The resulting tree was used to manually classify eukaryotic sequences into the following groups: α-tubulin, β-tubulin, γ-tubulin, δ-tubulin, ε-tubulin, and cryptic tubulins. We then downsampled these sequences by running CD-HIT (*45*) (-c 0.55 for α, β, and ε’ -c 0.6 for γ; and -c 0.7 for δ) to generate a balanced dataset. Separately, we recruited Asgard archaeal and verrucomicrobial (bacterial) BtubAB tubulins that clustered with eukaryotic tubulins and down sampled them separately using CD-HIT (-c 0.8) and sequences clustering with artubulins. To search for additional Asgard archaeal sequences published since our initial searches in 2023 (*13*, *46*–*48*), we combined the sequences in a single FASTA file, aligned it with MAFFT-linsi (*49*), trimmed with trimAl (-gt 0.5), and used the resulting alignment to perform one more search against NCBI nr (July 2025) and a local database of Asgard archaeal genomes using PsiBLAST (-num_iterations 1 -evalue 1e-10).

To obtain a well-populated outgroup, we used CetZ sequences from our previous searches, plus a public sequence dataset by Santana-Molina *et al*. (*8*) and aligned them using FAMSA, together with the set of eukaryotic, Asgard archaeal, and bacterial tubulins. We then trimmed this alignment using trimAl (-gt 0.5), removed gappy sequences (more than 50% of gaps), and aligned with FastTreeMP (-lg). In the resulting tree, we selected sequences form a clade representing CetZ and removed redundancy using CD-HIT (-c 0.65). To obtain a sequence dataset for artubulins, we selected identified artubulins from (*8*) and from our initial searches above, aligned them using MAFFT-linsi, used this alignment for an additional sequence similarity search against NCBI’s nr database (February 2025) using PsiBLAST (-num_iterations -evalue 1e-100), and removed redundancy using CD-HIT (-c 0.8). To investigate the effect of combining different sets of sequences, we used four datasets: one with all eukaryotic tubulins, Asgard archaeal tubulins, and bacterial tubulins; one adding artubulins; one adding CetZ; and one adding both artubulins and CetZ. For all these datasets, we aligned sequences using MAFFT-linsi, trimmed with trimAl (-gt 0.5 or -gt 0.1), and removed sequences with more than 50% gaps. We evaluated model fit using ModelFinder (*50*) within IQ-TREE v3.0.1 (*51*) (command: iqtree3 -m MFP -mset WAG+C40, LG+C40, Q.pfam+C40, Q.pfam_gb+C60, WAG+C50, LG+C50, Q.pfam+C50, Q.pfam_gb+C60, WAG+C60, LG+C60, Q.pfam+C60, Q.pfam_gb+C60 -mrate G4 -mfreq '' -n 0) and identified Q.pfam+C50+G4 as the best model according to the Bayesian information criterion in all instances. We then reconstructed phylogenies using IQ-TREE 3 under this model and an additional tree using the posterior mean site frequency (PMSF) approximation of this model (*52*) to reconstruct 100 standard bootstrap pseudoreplicates. Some alignments were additionally trimmed during the reconstruction process by removing the columns with the 10% lowest log-likelihood values (“--robust-phy 0.9” in iqtree3) (*53*). Transfer bootstrap expectation (*54*) was calculated using RAxML-ng (*55*). For visualization, trees were rooted using madRoot (*56*) and plotted using FigTree v1.4.4 (*57*). Maps to visualize the neighborhoods around tubulin homologs were plotted using genoPlotR (*58*) including homology links identified with DIAMOND v2.1.9.163 (*59*) sequence similarity comparisons under default parameters.

### Cloning and expression of AtubAB in *E. coli*

Metagenomic sequencing revealed an operon in Kariarchaeaceae 81MaySF_Bin13 (Fig. 1, A and B, and fig. S1C) that on the translated protein sequence level showed significant sequence similarity to eukaryotic tubulins and bacterial BtubAB. We named these proteins Kari AtubA and AtubB, and they were subsequently added by others to GenBank under accession numbers MDH5401500.1 and MDH5401501.1, respectively. AlphaFold 3 (*28*) confirmed the tubulin fold of AtubA and AtubB (fig. S1D) but also revealed that the amino acid sequence of AtubA as deposited in MDH5401500.1 is likely too short at the N terminus because the first β strand is not completing the central β sheet in the N-terminal domain of the tubulin fold. Inspecting the nucleotide sequence revealed that there is likely a rare ATT start codon upstream, and this produces the sequence MSEVVVV (replacing M1 in MDH5401500.1) that is related to other tubulins near the N terminus as confirmed by multiple sequence alignments. In the modified operon, the ATT start codon also positions a likely RBS in a plausible distance to the first start codon (fig. S1C). The resulting Kari *atubA* and *atubB* genes as used in this study are colored blue and red in fig. S1C. Expression of AtubAB in *E. coli* was facilitated by the construction of artificial bicistronic expression vectors. For this, the *atubA* and *atubB* genes were codon optimized for expression in *E. coli* [Integrated DNA Technologies (IDT)], and a larger intergenic region with its own (nonoverlapping) RBS was placed between them. Two constructs were made: AtubAB untagged and AtubAhBs, with AtubA His_8_ tagged and AtubB 2xStrep tagged [each tag preceded by a tobacco etch mosaic protease (TEV) cleavage site]. Overhangs for Gibson assembly into the expression vector pHis17 (for example, Addgene, plasmid #78201) were added to both constructs, resulting in the sequences listed in fig. S2 (A and B), which were provided by IDT as gBlocks. Gibson assembly with a linearized version of pHis17 by polymerase chain reaction using the primers CGATCCGGCTGCTAACAAAGCCCGAAAGGA and CATATGTATATCTCCTTCTTAAAGTTAAAC resulted in the two expression vectors, pHis17-AtubAB and pHis17-AtubAhBs, whose correctness was confirmed by Sanger sequencing. *E. coli* C41(DE3) cells were transformed with the two expression vectors, and expression levels were tested in 10 ml of 2xTY cultures containing 100 μM ampicillin at 37°C. After induction with 1 mM IPTG (isopropyl-β-d-thiogalactopyranoside), cells were grown further at 37°C for 4 to 6 hours, harvested and taken up in 1 ml of B-PER complete (Thermo Fisher Scientific), and incubated for 15 min at room temperature (RT) for lysis. Centrifugation at ×20,000*g* at 4°C separated soluble lysates from insoluble pellets.

### Expression and purification of His_8_- and Strep-tagged AtubAhBs

*E. coli* C41(DE3) cells were transformed with the pHis17-AtubAhBs expression vector and grown first in 200 ml and then 6 liters of 2xTY supplemented with 100 μM ampicillin at 37°C/200 rpm. After addition of 1 mM IPTG, cells were grown for another 5 hours at 37°C/200 rpm, harvested by centrifugation, and stored at −70°C. A total of 500 ml of buffer A [50 mM tris and 200 mM NaCl (pH 7.5)] with some deoxyribonuclease (DNase) I and six cOmplete protease inhibitor tablets (Roche) was added to the cells to resuspend. Cells were lysed by a single pass through a Constant Systems cell disruptor at 35 kpsi. Ultracentrifugation in a Beckman 45 Ti rotor at 35,000 rpm for 1 hour was used to remove insoluble material. The lysate was pumped through two HisTrap HP nickel columns (Cytiva) at 2 ml/min. Washing and elution were done at 7 ml/min using buffer A and buffer B [1 M imidazole (only), pH 7.0] and involved steps of 2, 5, 10, 30, and 100% buffer B. AtubAhBs eluted mostly in the 10% fractions, which were checked by SDS–polyacrylamide gel electrophoresis (SDS-PAGE) and Coomassie staining. Protein was precipitated by adding 100% (sat.) ammonium sulfate to 50% (sat.) final concentration. The precipitate was isolated by centrifugation at ×45,000*g* at 4°C for 20 min. The pellet was resuspended in 2 ml of buffer A and subjected to SEC using a Sephacryl S-200 16/60 (Cytiva) column in buffer A at 1 ml/min. Fractions were checked by SDS-PAGE, and those containing AtubAhBs were pooled and frozen into aliquots at −70°C. Final concentration (without concentrating) was 6 g/liter as determined with an ultraviolet (UV) spectrometer and a calculated extinction coefficient. More than 100 mg of protein were obtained. The identity of the proteins was confirmed by intact mass spectrometry after TEV cleavage overnight (0.35 g/liter of TEV protease): AtubAh: 49,035 Da (theoretical, no M1: 49,036 Da); AtubBs: 47,033 Da (theoretical, no M1: 47,034 Da). The uncleaved AtubAhBs product with the His_8_ and Strep tags still present was used in this study.

### Expression and purification of untagged AtubAB

*E. coli* C41(DE3) cells were transformed with the pHis17-AtubAB expression vector and grown first in 200 ml and then 6 liters of 2xTY supplemented with 100 μM ampicillin at 37°C/200 rpm. After addition of 1 mM IPTG, cells were grown for another 5 hours at 37°C/200 rpm, harvested by centrifugation, and stored at −70°C. A total of 500 ml of buffer C (20 mM tris, pH 8.0) with some DNase I and six cOmplete protease inhibitor tablets (Roche) was added to the cells to resuspend. Cells were lysed by a single pass through a Constant Systems cell disruptor at 35 kpsi. Ultracentrifugation in a Beckman 45 Ti rotor at 35,000 rpm for 1 hour was used to remove insoluble material. The lysate was pumped through a HiPrep Q XL 16/10 anion exchange column (Cytiva) at 10 ml/min. Proteins were eluted at the same speed with a gradient to 50% of buffer D (buffer C + 1 M NaCl) against buffer C. Fractions were checked by SDS-PAGE and Coomassie staining. Protein was precipitated by adding 100% (sat.) ammonium sulfate to 50% (sat.) final concentration. The precipitate was isolated by centrifugation at ×45,000*g* at 4°C for 20 min. The pellet was resuspended in 3 ml of polymerization buffer {BRB80 with 500 mM potassium glutamate: 80 mM Pipes, 1 mM MgCl_2_, 1 mM EGTA, and 500 mM potassium glutamate [pH 6.9 (KOH)]} and subjected to SEC using a Sephacryl S-200 16/60 (Cytiva) column in polymerization buffer at 1 ml/min. Fractions were checked by SDS-PAGE, and those containing AtubAB were pooled, concentrated with a Vivaspin 20 10-kDa molecular weight cutoff concentrator (Sartorius) to ∼30 g/liter (as determined by UV with calculated extinction coefficient), and frozen into aliquots at −70°C. More than 100 mg of protein was obtained. The identity of the proteins was confirmed by intact mass spectrometry: AtubA: 48,097 Da (theoretical, no M1: 48,098 Da); AtubB: 46,095 Da (theoretical, no M1: 46,095 Da).

### SEC with multiangle light scattering

SEC-MALS was performed using an Agilent 1200 series LC system with an online DAWN HELEOS II system (Wyatt Technology) equipped with a QELS+ module (Wyatt Technology) and an Optilab rEX differential refractive index detector (Wyatt Technology). Untagged AtubAB at 3 g/liter was injected onto a Sephadex S200 Increase HR10/300 (Cytiva) size exclusion column preequilibrated in polymerization buffer {BRB80 + 500 mM potassium glutamate; 80 mM Pipes, 1 mM MgCl_2_, and 1 mM EGTA [pH 6.9 (KOH)]}. The light scattering and protein concentration at each point across the peaks in the chromatograph were used to determine the absolute molecular mass from the intercept of the Debye plot using Zimm’s model as implemented in the ASTRA v7.3.2.19 software (Wyatt Technology). To determine interdetector delay volumes, band-broadening constants, and detector intensity normalization constants for the instrument, BSA (bovine serum albumin) was used as a standard before sample measurements.

### GTPase assay

GTP hydrolysis by untagged AtubAB was monitored through the detection of the release of inorganic phosphate with a malachite green assay, following a previously published procedure (*60*). Samples contained 25 μM protein (dimer: 2.35 g/liter) in polymerization buffer [BRB80 with 500 mM potassium glutamate, pH 6.9 (KOH)] at 25°C.

### Steady-state concentration determination

A total of 10 g/liter (94 μM dimer) of untagged AtubAB in BRB80 with 500 mM potassium glutamate polymerization buffer was diluted three times 1.5-fold in polymerization buffer in 200 μl of volumes. A total of 100 μl of samples was prespun at 50,000 rpm for 20 min at 20°C in a TLA-100 rotor (Beckman). A total of 2 mM GTP was added, and the samples were incubated for 30 min at RT. Samples were then centrifuged at 50,000 rpm for 20 min at 20°C in the same TLA-100 rotor (Beckman). The supernatants were withdrawn; pellets were washed with 50 μl of polymerization buffer (no nucleotide) and were resuspended in 100 μl of gel loading buffer over 1 hour. Samples from supernatants and pellets were prepared in gel-loading buffer (1:10 dilution) for SDS-PAGE and subsequent Coomassie staining. Band intensities were integrated with BioRad’s Image Lab software and plotted. What was determined could be called a critical concentration of nucleation, but because of the dynamic instability present, we used the term steady-state concentration.

### Pelleting assays

Figure S2E: A 2.3 g/liter (25 μM dimer) of AtubAB in BRB80 with 500 mM potassium glutamate polymerization buffer were assembled in the presence of 2 mM GTP with 27.5 μM of laulimalide, paclitaxel, zampanolide, discodermolide, epothilone, MAY, pironetin, gatorbulin, vinblastine, colchicine, podophyllotoxin, sabizabulin, nocodazole, and plinabulin or the DMSO control. After 2 hours at 37°C, samples were centrifuged at 60,000 rpm for 30 min at 37°C in a TLA-100 rotor (Beckman). The supernatants were withdrawn, and pellets were resuspended in same volume of polymerization buffer. Samples from supernatants and pellets were prepared in gel-loading buffer for SDS-PAGE. Figure 2G: As above but 6 g/liter (63 μM dimer) of prespun (at 50,000 rpm for 20 min at 20°C in a TLA-100 rotor Beckman) AtubAB was mixed with 50 μM MAY and incubated at RT for 30 min, before spinning at 50,000 rpm for 20 min at 20°C in a TLA-100 rotor (Beckman). The DMSO and compound reactions contained 1 mM GTP. Figure 2E: As Fig. 2G but 10 g/liter (94 μM dimer) of prespun (at 50,000 rpm for 20 min at 20°C in a TLA-100 rotor Beckman) AtubAB was mixed with 2 mM GDP or GTP or 0.5 mM GMPCPP (Jena Bioscience) and incubated at RT for 30 min before spinning at 50,000 rpm for 20 min at 20°C in a TLA-100 rotor (Beckman).

### Nucleotide content determination

A solution containing 25 μM (2.4 g/liter) of untagged AtubAB in BRB80 with 500 mM potassium glutamate polymerization buffer was used. The sample was sedimented (×100,000*g* for 40 min at 25°C), and nucleotides from supernatant and pellets were extracted with cold 0.5 N HClO_4_ (with guanosine as internal standard). After 10 min at 4°C, denatured protein was removed by centrifugation (×12,000*g* for 10 min at 4°C). Aliquots were neutralized by the addition of one-sixth volume of 1 M K_2_HPO_4_, 0.5 M acetic acid, and one-sixth volume of 3 M KOH. For high-performance liquid chromatography (HPLC) analysis of the nucleotide content, precipitated KClO_4_ was removed by centrifugation (×12,000*g* for 10 min at 4°C), and nucleotides were separated by isocratic reverse-phase ion pair HPLC (Supelcosil LC-18-DB) in buffer 0.2 M K_2_HPO_4_, 4 mM tetrabutylammonium, and 0.1 M acetic acid (pH 6.7) as described: (*61*). Two independent experiments were run and produced similar results.

### Maytansine binding affinity determination

Fluorescence anisotropy competition experiments and analyses were performed as previously described (*62*), with minor modifications. The binding constant of the fluorescent FcMaytansine (maytansine with fluorescein attached) probe to purified untagged AtubAB was determined by fluorescence anisotropy titration at 25°C in black Nunc 96-well, flat-bottom microplates and in a final volume of 200 μl. A total of 90 nM FcMaytansine in assay buffer [15 mM Pipes-KOH (pH 7.0) and 1 mM EGTA supplemented with 1.5 mM MgCl_2_ and 0.1 mM GTP] was titrated with increasing amounts of AtubAB up to 1 μM. The anisotropy and fluorescence values of the free (*r* = 0.035 ± 0.001) and bound (*r* = 0.256 ± 0.005) states were determined in the absence and presence of 1 μM AtubAB, respectively. The anisotropy was measured using an iD5 microplate reader (Molecular Devices). Samples were equilibrated at 25°C before measurement. Excitation and emission wavelengths were set to 485 and 435 nm, respectively. The read height and the gain were previously adjusted using both fluorescein and FcMaytansine. To calculate the binding constant of FcMaytansine, the fractional saturation, ν_b_, of the probe had to be determined. Binding of FcMaytansine to AtubAB was measured through changes in its anisotropy. The anisotropy of a mixture of free and bound FcMaytansine can be expressed as

$$r=F_{b}\times r_{b}+F_{f}\times r_{f}$$ (1)

where *r* is the measured anisotropy and *F*_f_ and *F*_b_ are the fractional fluorescence intensities of free and bound FcMaytansine, respectively. *r*_f_ is the anisotropy of the free FcMaytansine, and *r*_b_ is the anisotropy of the bound FcMaytansine. Because there is a 2.6 ± 0.2–fold difference between the fluorescence intensity of the bound and free FcMaytansine, the fluorescence fraction is weighted by the change in fluorescence intensity according to

$$F_{b}=\nu_{b}\times \left(I_{b}/I_{t}\right)$$ (2)

where *I*_b_ is the fluorescence intensity of bound FcMaytansine and *I*_t_ is the total fluorescence intensity. Given these two equations

$$r=\left(I_{f}/I_{t}\times \nu_{f}\times r_{f}\right)+\left(I_{b}/I_{t}\times \nu_{b}\times r\right)$$ (3)

where *I*_f_ is the fluorescence intensity of free FcMaytansine and ν_f_ is the fractional saturation of free FcMaytansine. Because the sum of the fractions of free (ν_f_) and bound (ν_b_) ligand is 1 and

$$I_{t}=I_{f}\times \nu_{f}+I_{b}\times \nu_{b}$$ (4)

Employing Eqs. 1, 3, and 4, we obtain the fractional saturation of FcMaytansine needed to calculate the binding constant of its binding to the site and of a ligand by competition with a probe of known binding constant

$$\nu b=\left(r–r_{f}\right)/\left(r–r_{f}\right)+R\;\left(r_{b}–r\right)$$ (5)

with *R* representing the ratio between the fluorescence intensity of the bound and free species (*R* = *I*_b_/*I*_f_). Once the fractional saturation of FcMaytansine in the experiments is known, the free concentration of AtubAB and FcMaytansine in the titration assay can be calculated from the difference between the bound and the total concentration of the probe. The free concentration of AtubAB was calculated from the difference in the total and bound concentration of FcMaytansine assuming a stoichiometry of 1:1. The values of fractional saturation of FcMaytansine bound versus the free AtubAB concentration were used to determine a binding constant at 25°C using SigmaPlot v13 (Systat Software).

Then, samples containing 90 nM FcMaytansine and 90 nM untagged AtubAB in assay buffer were titrated with increasing amounts of a competitor at 25°C in black Nunc 96-well, flat-bottom microplates and in a final volume of 200 μl. Fluorescence anisotropy measurements were carried out as described above. The binding constant of maytansine *K*_m_ can be determined from the known values of the binding constant of FcMaytansine *K*_Fc_ and the total concentrations of binding sites by solving the equations

$$K_{m}={\left[\text{maytansine}\right]}_{\text{bound}}/{\left[\text{sites}\right]}_{\text{free}}\times {\left[\text{maytansine}\right]}_{\text{free}}$$ (6)

$$K_{\text{Fc}}={\left[\text{FcMaytansine}\right]}_{\text{bound}}/{\left[\text{sites}\right]}_{\text{free}}\times {\left[\text{FcMaytansine}\right]}_{\text{free}}$$ (7)

$${\left[\text{FcMaytansine}\right]}_{\text{free}}={\left[\text{FcMaytansine}\right]}_{\text{total}}–{\left[\text{FcMaytansine}\right]}_{\text{bound}}$$ (8)

$${\left[\text{maytansine}\right]}_{\text{free}}={\left[\text{maytansine}\right]}_{\text{total}}–{\left[\text{maytansine}\right]}_{\text{bound}}$$ (9)

$${\left[\text{sites}\right]}_{\text{free}}={\left[\text{sites}\right]}_{\text{free}}–{\left[\text{maytansine}\right]}_{\text{bound}}–{\left[\text{FcMaytansine}\right]}_{\text{bound}}$$ (10)

The system was solved using EQUIGRA5 (*62*) using the binding constant of FcMaytansine and a stoichiometry of 1:1 for the AtubAB-FcMaytansine complex

### 90° light scattering assays

Ninety-degree light scattering was measured on a Cary Eclipse fluorescence spectrometer (Agilent Technologies). A nonstirred cuvette was used: 10-mm 28F/MS-Q10 (Spectrology), taking 900 μl of sample. Settings were as follows: 360-nm excitation and emission wavelengths, 23°C reaction temperature, 5-nm slits, 1-s averaging, photomultiplier setting low, and 700-min total collection time. A 6 g/liter (64 μM dimer) of tagged AtubAhBs or untagged AtubAB in BRB80 with 500 mM potassium glutamate [pH6.9 (KOH)] polymerization buffer was used in 900 μl volumes. A total of 44 μM of nucleotides was used to start the reactions. MAY at 10 mM in DMSO was added to 200 μM final concentration.

### FIB milling for lamella production

To produce samples of sufficiently thin ice for STA, grids of *E. coli* C41(DE3) cells overexpressing untagged AtubAB (plasmid pHis17-AtubAB) were prepared for cryo-FIB milling. Cells were grown in 2xTY medium to optical density at 600 nm (OD_600_) ∼ 1 before induction with 1 mM IPTG for several hours until > OD_600_ of 3 was reached. Cells were pelleted at ×1800*g* for 5 min before resuspending in fresh medium to OD_600_ ∼ 60 (viscous). Quantifoil Au R2/2 200 mesh grids were glow discharged using a PELCO easiGlow at 25 mA for 45 s. Samples were plunge frozen using a Vitrobot Mark IV (Thermo Fisher Scientific) at 21°C with humidity control turned off. A total of 8 μl of concentrated *E. coli* sample was applied to the front faces of the grids, which were then back blotted for 6 s (blot force of 10) with the aid of a custom-made Teflon ring in the shape of a Vitrobot blot paper. Plunge-frozen grids were then clipped and loaded onto a Thermo Fisher Scientific Aquilos 2 Dual Beam Cryo-FIB/SEM for automated milling and polishing. Lamella positions were assigned using MAPS software (Thermo Fisher Scientific), while milling was performed using the AutoTEM software (Thermo Fisher Scientific) with our own optimized milling procedure. Grids were coated with a layer of organoplatinum using a gas injection system for 40 s, followed by 30 s of sputtering with inorganic platinum to reduce charging effects during milling. A milling angle of 12° was chosen for all sites. Milling of lamellae using the gallium beam (all at 30 kV) was fully automated, aside from initiation of the final polishing step, and was carried out in several steps of incrementally reduced milling currents and lamella widths. Rough milling was carried out at 1 nA (rectangular pattern) with a starting lamella width of 10 μm, followed by successive steps at 0.3 nA, 0.1 nA, and 50 pA. Each site was milled “site wise” (as opposed to “step wise”) overnight, ensuring that up to 30 lamellae were milled up to the final polishing step in the morning. A final polish at 30 pA was performed the following morning for the best 20 lamellae, ensuring that samples remained as freshly polished as possible before transferring out of the Aquilos 2 Cryo-FIB/SEM. Final lamella thicknesses were targeted to 110 nm, with a final width of 6 μm. See fig. S3A for actual lamella thicknesses as determined by subsequent cryotomography.

### Cryo–electron tomography

Lamellae prepared by cryo-FIB milling were transferred to a Thermo Fisher Scientific Krios G4 300 kV Transmission Electron Microscope (TEM), equipped with a Falcon 4i detector, a Selectris X energy filter, and a cold field emission gun. Data were collected using the Tomography 5 software (Thermo Fisher Scientific), with search maps of lamellae used to target regions for collection. A magnification of ×83,000 (1.514 Å per pixel) was used to collect tilt series bidirectionally. A starting stage tilt angle of 12° followed by 3° tilt increments to −42° (for the first branch) before completing the second branch from 12° to 66°. The samples were exposed for 1.27 s across eight frames per tilt image, with a dose accumulation of 4.1 e^−^/Å^2^ per tilt or 151.7 e^−^/Å^2^ across all 37 tilts. An objective aperture of 100 μm and an energy filter slit width of 10 eV were selected and centered immediately before data collection. A target defocus range of −1.5 to −3.5 μm was applied across the data collection.

### STA of AtubAB mini microtubules in *E. coli*

Tilt series data were imported into the Relion 5 STA pipeline (*33*). Tilt series were motion corrected using Relion’s implementation of MotionCor2 (*63*) with 5 × 5 patches, followed by contrast transfer function (CTF) estimation with CTFFIND-4.1 (*64*). Bad tilts were manually removed using the Napari-based viewer (*65*, *66*) in Relion 5 before tilt series alignment in Relion 5 with AreTomo2 (*67*) using an estimated tomogram thickness of 120 nm and correcting for tilt angle offset (because of the FIB milling angle). Tomograms were reconstructed with unbinned pixel dimensions of 4000 × 4000 × 2000 at 10 Å per pixel and denoised using CryoCARE (*68*). For all tomograms, the sample thicknesses were measured to be at or below 200 nm using the software Geollama (*69*). Filament picking was carried out on denoised tomograms using the Relion 5 Napari picking viewer, allowing annotation of filament paths. Particle poses were determined using “relion_python_tomo_get_particle_poses” with unknown filament polarity. All subsequent processing steps used Relion 5 2D tilt stacks for particles. Initial poses were extracted at bin 3 (4.542 Å per pixel), with a maximal dose accumulation of 60 e^−^/Å^2^ and a box size of 70 binned pixels (318 Å). Because of the uncertainty around the filament composition (number of protofilaments), an initial model-free helical refinement against a cylinder without imposed symmetry was performed, revealing a four-stranded filament. With the subunit spacing along the helical axis being ∼40 Å (the canonical longitudinal tubulin repeat), a helical rise of 10 Å was estimated to allow for four subunits per 40 Å. With the direction of the twist unknown, two refinements were conducted in tandem against a cylinder, one with a 90° twist and another with a −90° twist. The negative twist refinement presented with a better final map and a substantially better angular accuracy. Thus, a final “initial” refinement was performed on the extracted coordinates with a −90° twist and 10-Å rise using the negative twist map as a reference (low-pass filtered to 30 Å). A subsequent masked refinement with symmetry searching was performed, yielding a map with an estimated resolution of 9 Å (Nyquist limit at bin 3) and helical parameters of −89.86° twist and 10.29-Å rise. These particles were then reextracted at bin 2 with a box size of 106 pixels (321 Å) and a maximum dose accumulation of 60 e^−^/Å^2^. Another masked refinement was performed following removal of duplicate particles resulting in an 8.22-Å resolution map [gold standard Fourier shell correlation (FSC) cutoff of 0.143]. 3D classification was performed to remove suboptimal particles, leaving 16,000 particles for another round of 3D refinement, this time with Blush regularization (*70*). This resulted in a map with an estimated resolution of 7.5 Å. These particles were then extracted at bin 1 for a round of Relion 5 Tomo refinement cycles consisting of CTF refinements and Bayesian polishing. Following an improvement in resolution from each step, a new bin 1 volume was reconstructed to benefit from the improvements gained. Polishing was performed with per-particle motion switched on and per-frame 2D deformation estimations. These steps improved the resolution of reconstructed volumes to 6.4 Å (FSC 0.143 criterion). Further refinements and classifications did not improve resolution much more, and so the final map chosen was that reconstructed from the last round of Bayesian polishing. Local resolution was estimated in Relion 5, and the final reconstruction was sharpened using EMReady (*71*). See fig. S3D for an overview of STA processing.

### Negative staining EM

Untagged AtubAB in polymerization buffer [BRB80 with 500 mM potassium glutamate, pH 6.9 (KOH)] was diluted to 6.6 g/liter with polymerization buffer, and 3 mM GTP (or 3 mM GDP or 0.6 mM GMPCPP) was added. After 1 hour of incubation at RT, the sample was rapidly diluted to 0.3 g/liter with polymerization buffer, and 5 μl was applied to 50-s glow-discharged, carbon-coated EM grids (5- to 6-nm amorphous carbon film, 400 mesh copper, CF400-CU, Electron Microscopy Sciences) and incubated for 30 s. After blotting off the protein solution with filter paper, 5 μl of 2% (w/v) uranyl acetate solution was applied three times and blotted off immediately with filter paper each time. Images were taken at various magnifications on a Philips Tecnai T12 Spirit, operated at 120 kV, and equipped with a Gatan Orius charge-coupled device detector.

### Cryo-EM (SPA) sample preparation

For AtubAB filaments, untagged AtubAB in polymerization buffer [BRB80 with 500 mM potassium glutamate, pH 6.9 (KOH)] was diluted to 7 g/liter with polymerization buffer, and 4 mM GTP was added. After incubating the sample at RT for 1 hour, the sample was further diluted to 0.25 g/liter with polymerization buffer and used immediately. For vitrification, a Leica EM GP2 automatic plunger was used. Temperature for plunging was 18°C, humidity was 99% (relative), and a blot time of 5 s was used. A total of 3.5 μl of sample was added to 60-s glow-discharged Quantifoil Au R2/2 200 mesh grids and blotted before vitrification in liquid ethane kept at −180°C. For the unpolymerized AtubAB heterodimer, untagged AtubAB in polymerization buffer was diluted to 0.5 g/liter in 50 mM Hepes, 100 mM KCl, 5 mM Mg-acetate, and 1 mM EGTA [pH 7.7 (KOH)]. For vitrification, a Vitrobot Mark IV (Thermo Fisher Scientific) automatic plunger was used. Temperature for plunging was 8°C, humidity was 100% (relative), and a blot time of 3.5 s at blot force of 10 was used. A total of 3.5 μl of sample was added to 60-s glow-discharged Quantifoil Au R1.2/1.32 300 mesh grids and blotted before vitrification in liquid ethane kept at −180°C.

### Cryo-EM (helical SPA) structure determination of AtubAB mini microtubules

All processing of micrographs was performed in Relion 5 (*72*) unless otherwise stated. A total of 4349 movies was collected at 1.222 Å per pixel using the EPU software (Thermo Fisher Scientific) on a Krios G4 microscope equipped with a Falcon 4i camera, a Selectris X energy filter, and a cold field emission gun (Thermo Fisher Scientific), operated at 300 kV. The movies were combined and motion corrected using Relion’s own implementation before CTF estimation using CTFFIND4 (*64*). Because single filaments were rare (because of bundling), manual picking was used to produce 61,000 particles, extracted 42 Å along each filament in boxes of 266 pixels. Initial helical parameters were determined using cryoSPARC 4 (*73*) helical refinement without providing helical parameters or a model. This produced a clear four-stranded density without difficulty, with the helical parameters twist = −89° and rise = 10.5 Å. These parameters describe an arrangement that must be pseudohelical for tubulin-like heterodimers because the rise per near-full turn (−356°) is 42 Å, which is the same distance from one subunit in an alternating AtubAB (…ABABAB…) protofilament to the next (A to B) and not to the same type, two subunits away (A to A). This means that the structure must have a seam where A contacts B and vice versa (see Fig. 2G for a schematic of this). In other words, helical processing with the parameters determined will average A and B subunits onto each other. Obtaining such an averaged map was done first using Relion 5 helical Refine3D with the helical parameters. Subsequent Class3D, CTFRefine, and Polish jobs, followed by another round of helical Refine3D, produced a fully symmetrical (A and B not distinguished) density at 3.1-Å resolution (twist = −89.84°, rise = 10.46 Å; FSC criterion 0.143). To resolve the A and B subunits in the map, resolving the eightfold redundancy (fourfold around the tube and twofold along the tube) was tried by first symmetry expanding eightfold with Relion 5’s relion_particle_symmetry_expand command, with the helical parameters, and subsequent Class3D without alignment into different numbers of classes and different *T* values—this failed. Instead, Class3D without alignment, with a mask around a single dimer, succeeded, as indicated by the finding that the one major difference in structure between AtubA and AtubB that AlphaFold 3 predicted (residues 102 to 111) was resolved almost completely (Fig. 2J). This did not resolve the seam position but makes it possible to demonstrate that the structure solved is made of AtubA and AtubB in alternating protofilaments. The symmetry dictates that such a structure must have a seam. Because the Class3D job is not gold standard, phenix.auto_sharpen (*74*) was used to produce a final density for the heterodimer in the AtubAB filament. Relion’s ModelAngelo (*35*) was used to build atomic models into the map, which went well, completely and automatically building AtubA on top and AtubB at the bottom of the dimer without human intervention. The model was checked and refined further with rounds of refinement with phenix.real_space_refine and manual building in MAIN (*75*). Phenix.model_vs_map reported a final model to map resolution of 3.1 Å (FSC criterion 0.5). To obtain a complete atomic model, the AtubAB mini microtubule, four refined dimers were placed in the symmetrized map and symmetry expanded with the symmetry twist = 1.31° (8 × –89.84° + 720°), rise = 83.68 Å (8 × 10.46 Å). For an overview of the helical cryo-EM processing, please consult fig. S3D.

### Cryo-EM (SPA) structure determination of unpolymerized AtubAB

All processing of micrographs was performed in Relion 5 (*72*). A total of 10,328 movies was collected at 0.955 Å per pixel using the EPU software (Thermo Fisher Scientific) on a Krios G4 microscope equipped with a Falcon 4i camera, a Selectris X energy filter, and a cold field emission gun (Thermo Fisher Scientific), operated at 300 kV. The movies were combined and motion corrected using Relion 5’s own implementation before CTF estimation using CTFFIND4 (*64*). Particles were picked using Topaz (*76*), and its general model produced ∼1 million particles, which were extracted in boxes of 210 pixels. Multiple rounds of Class2D reduced particle numbers to 380,000, revealing clear side and top views of the AtubAB heterodimers (fig. S3H). Refine3D with a heterodimer reference model (AtubAB filament structure as determined above) filtered to 20-Å resolution produced a good map that revealed secondary structure elements but also highlighted that parts of AtubA were disordered. Rounds of Class2D and Class3D without alignment reduced particle numbers to 190,000, at which point CTFRefine and Polish, followed by Refine3D produced an even better map. One round of Class3D without alignment found 125,000 good particles that, after a last Refine3D and Postprocess, produced the final heterodimer density at 3.5-Å resolution (FSC criterion 0.143). The previously obtained AtubAB heterodimer atomic structure was placed in the density and refined and adjusted with rounds of phenix.real_space_refine (*74*) and manual building in MAIN (*75*). For an overview of the processing, please consult fig. S3I.

### AtubAB labeling for TIRF microscopy

AtubAB filament labeling with fluorescent dyes was performed by adopting protocols used for bacterial BtubAB previously (*11*). Aliquots of AtubAB were thawed and diluted to 40 μM (dimer: 3.8 g/liter) in polymerization buffer {for TIRF only: 80 mM K-Pipes, 500 mM potassium glutamate, 1 mM EGTA, 5 mM MgCl_2_, and 2 mM GTP [pH 6.9 (KOH)]} and incubated at RT for 40 min to allow for filament polymerization. The reaction was centrifuged at ×100,000*g* in a TLA-120.2 rotor (Beckman) for 10 min at 20°C, the supernatant was discarded, and the pellet was resuspended in polymerization buffer, adjusting the AtubAB concentration to 40 μM. For fluorescence labeling, the filaments were then used to resuspend a full tube of either Alexa Fluor 647 *N*-hydroxysuccinimide (NHS) ester (Invitrogen, A20006) or Atto 488 NHS ester (Sigma-Aldrich, 41698-1MG-F). For biotin labeling, ∼2 mg of EZ-Link Sulfo NHS-LC-LC-Biotin (Thermo Fisher Scientific, 21343) were resuspended in polymerization buffer and added to the filaments at a final concentration of 400 μM (10-fold molar excess). The reactions were incubated at RT for 30 min with gentle rocking and then quenched by adding 1 mM tris-HCl (pH 7.0) and incubating for 5 min at RT. After incubation, the samples were transferred into polycarbonate ultracentrifuge tubes (Beckman), prefilled with 150 μl of cushion buffer [polymerization buffer supplemented with 60% (v/v) glycerol]. Cushion buffer was used to ensure the removal of the nonreacted free dye. The tubes were then centrifuged at ×100,000*g* in a TLA-120.2 rotor (Beckman) for 10 min at 20°C. The supernatant was discarded, and the pellet was resuspended (depolymerized) in glutamate-free buffer {80 mM K-Pipes and 1 mM EGTA [pH 6.9 (KOH)]}. The filaments were incubated at RT for 1 hour to ensure their depolymerization. Subsequently, the reaction was centrifuged again at ×100,000*g* in a TLA-120.2 rotor (Beckman) for 20 min at 20°C. The supernatant was recovered; the protein concentration and the labeling efficiency were estimated using a NanoDrop spectrometer (Thermo Fisher Scientific) and calculated extinction coefficients. The proteins were mixed to make two different stocks: AtubAB seed stocks were prepared at 50 μM final concentration (20% fluorescent-AtubAB and 10% biotinylated-AtubAB) in polymerization buffer supplemented with 5 mM GMPCPP (Jena Bioscience, NU-405S); free (unpolymerized) AtubAB dimers were prepared at a final concentration of 50 μM (20% fluorescent-AtubAB) in glutamate-free buffer. Both solutions were aliquoted in 2 μl, immediately flash-frozen after mixing, and stored for future experiments. Tagged AtubAhBs was added unlabeled.

### AtubAB TIRF microscopy

Glass coverslips (22 × 22 mm; NEXTERION, Schott) were incubated for at least 48 hours at RT with gentle agitation in a 1:10 (w/w) mix of mPEG-Silane (30 kDa; PSB-2014, Creative PEGWorks) and PEG-Silane-Biotin (3.4 kDa; Laysan Bio) at a final concentration of 1 g/liter in 96% (v/v) ethanol and 0.2% (v/v) HCl. On the day of each experiment, the coverslips were washed with ethanol and ultrapure water, dried with a nitrogen gas gun, and assembled into an array of flow cells on mPEG-Silane passivated slides using double-sided tape (Adhesives Research ARseal 90880; precisely cut with a Graphtec CE6000 cutting plotter). The chamber was first perfused with BRB80 buffer {TIRF only: 80 mM K-Pipes, 1 mM EGTA, and 5 mM MgCl_2_ [pH 6.9 (KOH)]}, and then NeutrAvidin (25 μg/ml; Thermo Fisher Scientific) was added to the chamber, incubated for 5 min, and then washed out with 10 chamber volumes of BRB80. In the meantime, an aliquot of AtubAB seeds was thawed, 2 μl of polymerization buffer [BRB80 + 500 mM potassium glutamate, pH 6.9 (KOH)] was added, and the reaction was incubated at room temperature for 5 min to allow for filament polymerization. The reaction was then diluted to 30 μl in imaging buffer {80 mM K-Pipes, 200 mM potassium glutamate, 1 mM EGTA, 5 mM MgCl_2_, 2 mM GTP, potassium casein (0.1 g/liter), BSA (0.1 g/liter), 40 μM dithiothreitol, 64 mM d-glucose, glucose oxidase (160 μg/ml), and catalase (20 μg/ml) [pH 6.9 (KOH)]} and added to the chambers. After 5 min, the chamber was quickly washed with 50 μl of imaging buffer, and dynamic filaments were elongated from the seeds by injecting into the chamber a solution of 20% labeled AtubAB at a final concentration of 4 μM, supplemented with 5 mM GTP. Experiments with AtubAhBs were performed following the same procedure, but the final concentration in the chamber was 5 μM, composed of 20% fluorescent-AtubAB, 60% unlabeled AtubAB, and 20% AtubAhBs. The microscope stage was kept at RT. Images were collected every 5 to 10 s in a custom spinning disk/TIRF microscope previously described in (*77*).

## Supplementary Materials

### The PDF file includes:

Figs. S1 to S16 Table S1 Legends for movies S1 to S5 Supplementary Discussion
