# Supplementary material for: Eukaryotic-like microtubules and dynamic instability of Asgard archaeal tubulins
Source: Sci Adv. 2026 Jul 23;12(30):eaeh1082. doi: 10.1126/sciadv.aeh1082 (PMC13394414; doi:10.1126/sciadv.aeh1082)
Supplement: Supplementary file 1 — Figs. S1 to S16 Table S1 Legends for movies S1 to S5 Supplementary Discussion [file sciadv.aeh1082_sm.pdf]

Science Advances
AAAS

Supplementary Materials for

Eukaryotic-like microtubules and dynamic instability of Asgard archaeal tubulins

Jan Löwe *et al.*

Corresponding author: Jan Löwe, jyl@mrclmb.ac.uk; Daniel Tamarit, d.tamaritchulia@uu.nl; Thijs J. G. Ettema, thijs.ettema@wur.nl

Sci. Adv. **12**, eaeh1082 (2026)
DOI: 10.1126/sciadv.aeh1082

The PDF file includes:

Figs. S1 to S16
Table S1
Legends for movies S1 to S5
Supplementary Discussion

Other Supplementary Material for this manuscript includes the following:

Movies S1 to S5

SUPPLEMENTARY FIGURES

Figure S1A. Full phylogeny corresponding to Figure 1A. The full tree is shown twice, with Transfer Bootstrap Expectation scores as branch support values shown on the left and Felsenstein Bootstrap Proportions on the right.

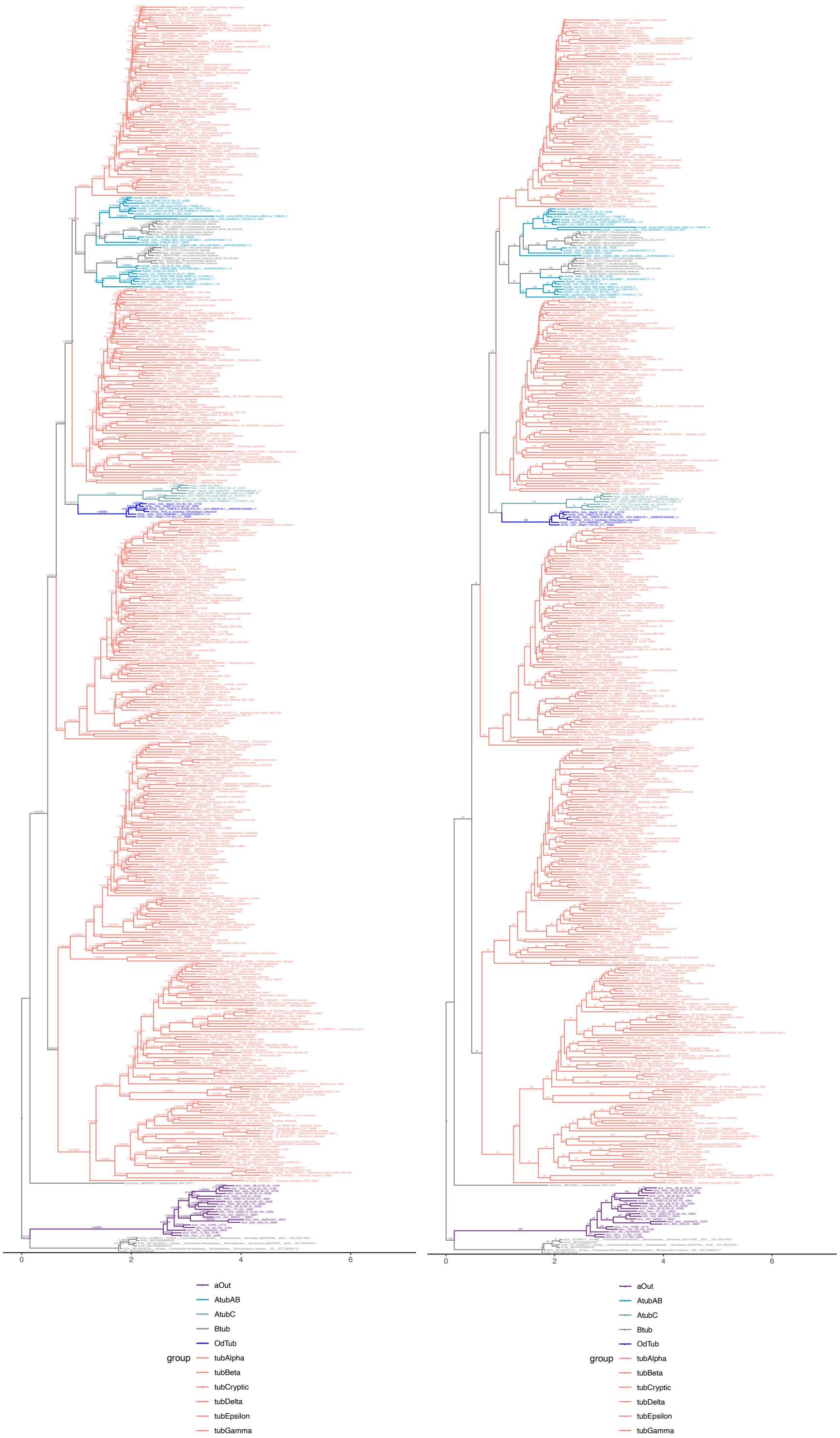

Figure S1B. Gene maps of Asgard archaeal tubulins and their genomic regions (7 kb up and downstream). Genes are represented with arrows, and dark vertical lines indicate contig boundaries. Comparison lines indicate homology as identified by Diamond sequence similarity searches (*59*).

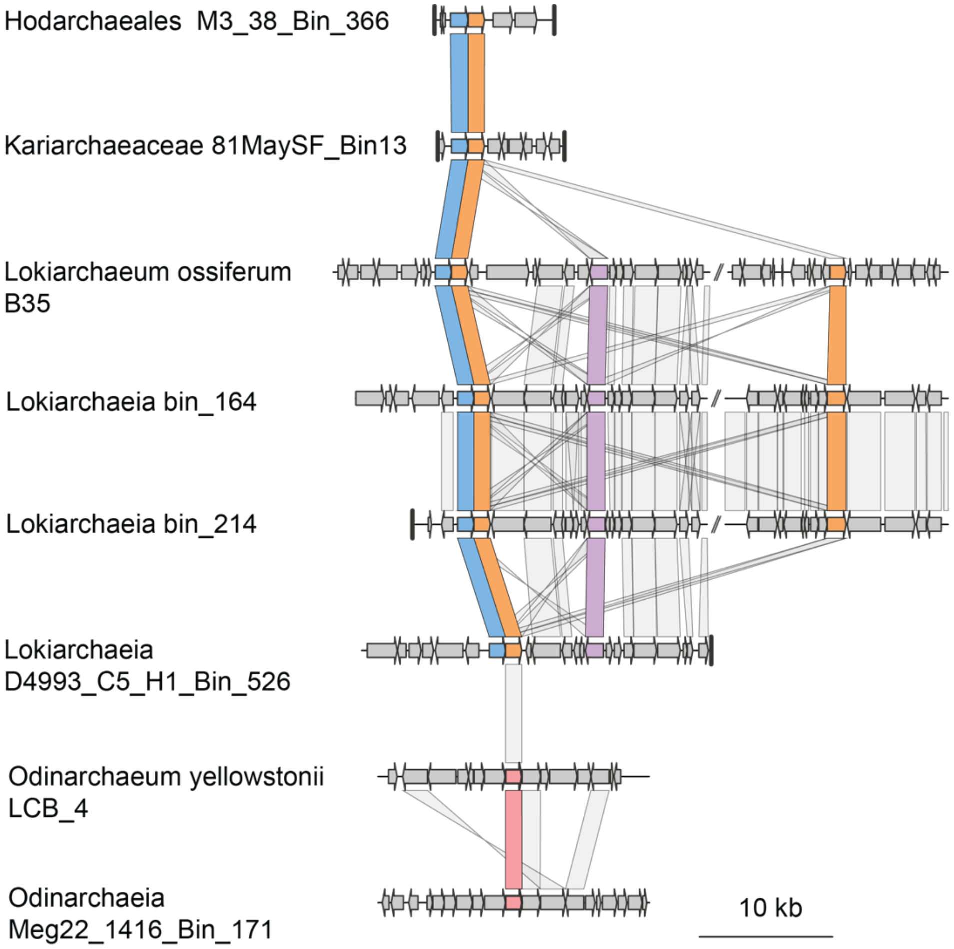

Figure S1C. **Kariarchaeaceae 81MaySF_Bin13 AtubAB operon metagenomic sequence** (as shown schematically in Figure 1B). Note that *atubA* was extended upstream towards the rare ATT start codon because the deposited predicted protein sequence (GenBank: MDH5401500.1) is too short for the tubulin fold (as determined with AlphaFold 3), where the residues missing from the deposited sequence are needed to form the first β strand of the protein. The start codon was previously misassigned, most likely. Blue: *atubA*, red: *atubB.* Putative ribosome binding sites (RBSs) have been underlined.

ATAACTGGAGGATATGACATATTTCAGAAGTCGTAGTAGTGGCCGTTGGTCAAGCCGGAAATCAAATTGCCGACCAATTTTGGCAGTTGATCGCTCAAGA
ACATAAAATTGGTCTAGATGGCATGCCAACCAATGGAGAGCCAACTGGAAGAACTGATGTTTTTTTCAGGAAGGTAAGCAATAGGTATGTTCCTAGAGCA
GTATTGGTTGATTTAGAACCTGCTGTATTGAAGAGTGTATCTGATGACGCCAAACCAAACTTCTATGATCCTAAAAACAAGGTTCATGGAGCAGATGGTG
CTGGAAACAACTTTGCTGTTGGGCATACCGAAATGGGTGCACAATATTTAGAACAAGTGATTGGAAAAATAAAAAACGAGGTAGAAAAAACTGATTCATT
GGGTGGATTTATAGTGACTCACAGTGTTGGTGGTGGTACTGGAAGTGGTTTCGGAACCTTAATTATGCAAAACATAAGAAAAGAATACCCAAATACACCT
TTACTATCATTTTCTATTTATCCAAGTCCAAGGATTAGTGATGTACTAACAGAACCTTACAATGCAATTTTTTCATCATACCGATTAGTAAGAGATACCA
GTTGTTCAATAATATATGATAATGATGCATTATACACAATATGCCAAAATCTCGGCATTGCAGAACCAACATATAGTGATTTGAACAAACTAATCTCTCA
AAGTATGGTAAACATAACTGCAGGAATGAGATTTGTTGGTACACTAAATCTTGATTTAAGGAAGTTAATAACAAACTTAGTTCCTCATCCAAGACTTCAT
TTCTTAATGACATCTACCGCACCTCTGGTAGAATCTGGTGGTTTTGATTCACAAAGTATTGAGAAAACAGCAGAAGTATTATTTGAGAAATCAAATATTC
AAGCTCAAGTTAATACTGAAGATGGTGCTTATATTTCAACAACAGTGTTGTTCAGAGGAAATGTAAATCCTGCAGATATGGATACTGCTTTACGTGCAAT
GAAGCAGAAATTGAAATTCGTATCATTTATACCAACTGGTTTTAAAACAGGTCTTTCTGCAACTGCACCTAATAATATGGAATTTGCAGCAGCATTAGTT
GCTAATCACACAGGTATAACTCAGGTATTTAAGAGAATTCTTGGCCAATTTGATAAATTATGGTCTAGGAAAGCTTTTTCACACTGGTATACTGATTCTG
GCTTAACAGAAGAAGATATTGTGAATGCAAGAAATGATATTGCTAAATTGGTTGAGGAATATGAGGAAACTTTGAAATTCAAAAGTAAGGATAAATCGGG
AGAGGAAGTAGCTGCAGCTGAAATTGCAGAAAATAAATAGGAGGAATAAGATAATGTCAAGAGAAATTATATCTATTCATGTTGGTCAGGCAGGGCTTCA
AGTCGGTGCAGCATTTTGGAGAACAGTTTTAGAAGAACACGGACTTGGTAATGATGGTTCTGTAGTTGATGCATCAAAAGAAAAATCAGCAGTTTATTTT
AAACAAGCTGGTAATAATAAGTTTGTACCTCGTGCAATTCTTGTAGATTTAGAACCAATGGTTATTAAAACAATCATGAATGATCTACCTGGATTATTCG
ATCCAGCTTACACCATTCATGGTCAATCCGGTGCTGCAAATAATTGGGCAAGAGGTTATCGAGGTGCTGATGGAGAAGTCAATGTTGATGATGTTTTAAA
GATTGTTGAAAAAGCAGTTTCAGAAACAGATAGTCTTCAAGGATTTTCAATCAGTCATTCTATTGGAGGTGGAACAGGATCTGGTCTTGGATCTCAAATT
TTGAAGAAATTAAAAGAAACATATCCAAAATATCCAATTATCACATTTAGTGTATTTCCCAGTCCATTGATATCTGATGCAGTTACTGAACCTTATAATT
CAGTATTTGCACTAGATCATTTGATTGAATATGCAGATGAAACTATTGTTTTGGATAACCATGCATTATACAACTTAACCAAAAACAACATGGGAATCAA
ATCTCCAAGCTATGCTGATTTAAACAAAATTATCTCTTGGGTCATGTCTGGTGTTACAGCATCCCTAAGATTTAAAGGTGATTTAAATACTGATCTCAAG
GAATTACTTGTTAATCTAGTACCATTCCCAAGACAACACTTCTTAACTTCAACATTCAGTCCAATAAAAAATGGAACTGGGTCTTTTGAAGAAGTTACAA
CTCATGATATCATAAATCAATTGTTTGATCCAAACAATGCAATGAGTGATACAAATTTTGATGAAGGTGCTTACATTGCATCTGTAGTTATGATGAGAGG
ACATGAAGTTAAGACAAATGCTGTCAATAGTTCATTAGCAGAGATAAAAGGTAAACTACGATTTTCAGAATTCATACCTACTGGAATTAAGACCGGTGTA
ACCTCTGAAACTTTAGTTGGATTTGAATCTACAGGGTTAAATTTATCGAATCATACAGGGATAACTAAAGTGTTCAAAAGATTAGCCGCACAATTTGATG
CTATGTATGAAAGAGATGCTTTCGTTCATTGGTATCTTGGCGAAGGAATGTCAAAAGATGATATGGAAAAAGCCAGAGAAAATCTAGGTAAGTTAACTAC
CGAATATGATACTAGTGTGTAATGACTTAGATAAAAATAAAA

Figure S1D. AlphaFold 3 prediction of AtubAB and stable heterodimer formation. AlphaFold 3 predicts AtubAB to be of the tubulin fold and to form heterodimers, and alternating ABAB protofilaments. Inspecting the AtubA and AtubB T7 loops in such a protofilament reveals that likely only one GTPase site is active, the one in AtubA (blue).

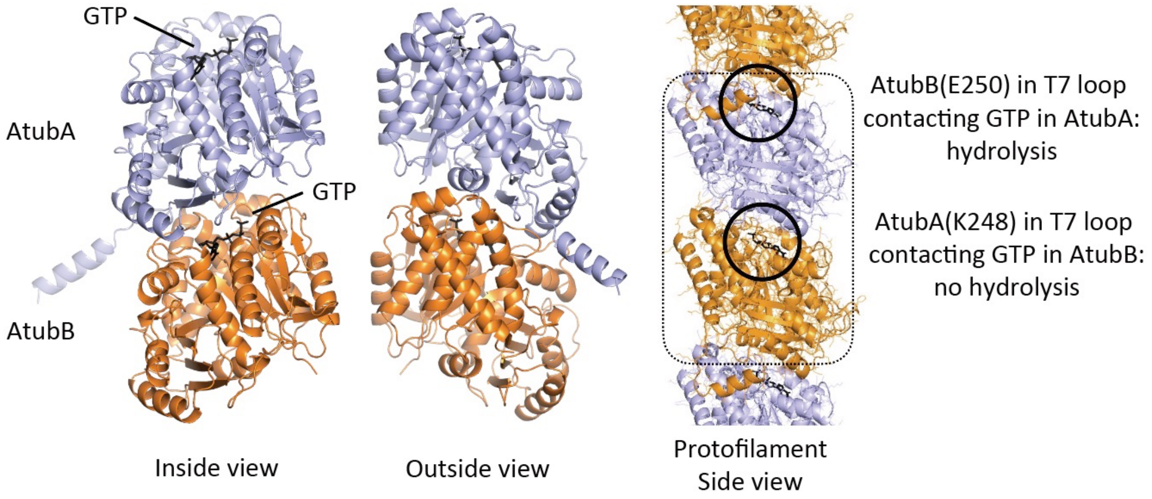

Figure S2A. AtubAB His$_8$- and Strep-tagged expression construct (AtubAhBs), codon optimised for expression in *E. coli*. Blue: *atubAh*, red: *atubBs.* Sequence of the gBLOCK used to make expression plasmid pHis17-AtubAhBs. RBSs have been underlined.

GTTTAACTTTAAGAAGGAGATATACATATGTCTGAAGTCGTTGTTGTCGCGGTGGGCCAAGCTGGTAATCAGATTGCGGATCAGTTTTGGCAGCTTATTG
CCCAGGAACATAAGATTGGCCTGGACGGAATGCCTACGAACGGTGAGCCTACGGGGCGCACCGATGTCTTTTTTCGTAAAGTATCCAACCGTTACGTACC
CCGCGCGGTGTTGGTCGACCTTGAGCCGGCTGTGCTGAAGTCTGTCAGTGACGATGCCAAGCCGAATTTCTACGACCCGAAGAACAAAGTGCACGGGGCC
GATGGCGCCGGAAATAATTTTGCGGTGGGTCACACGGAAATGGGAGCGCAGTACTTGGAACAAGTAATTGGCAAAATCAAAAACGAGGTAGAGAAAACCG
ACTCATTGGGTGGGTTTATTGTTACACACAGTGTTGGAGGTGGTACCGGTTCTGGATTTGGAACTTTGATCATGCAGAATATCCGTAAAGAATATCCAAA
CACTCCGCTTTTGAGTTTTTCAATTTACCCCTCCCCCCGTATCTCCGATGTGCTGACCGAGCCATATAATGCGATCTTCAGTAGTTATCGCTTAGTGCGT
GACACATCATGCAGTATTATTTACGACAATGACGCATTGTATACCATCTGTCAGAACTTAGGGATTGCAGAACCAACATACTCTGACTTGAACAAGCTTA
TTTCACAAAGTATGGTCAATATCACCGCGGGTATGCGCTTTGTTGGTACCTTAAACCTTGACCTTCGCAAACTGATTACAAACTTGGTACCCCATCCGCG
TTTACACTTTCTTATGACTAGTACGGCACCGCTGGTAGAATCAGGTGGTTTCGATTCGCAATCCATTGAAAAAACGGCCGAGGTCCTGTTTGAAAAATCG
AATATCCAAGCTCAGGTGAATACGGAAGATGGCGCCTATATTTCAACAACCGTTTTGTTTCGCGGGAACGTCAACCCAGCTGACATGGATACTGCTCTTC
GTGCAATGAAGCAAAAGCTGAAATTTGTCTCGTTTATTCCGACCGGTTTCAAGACAGGACTGTCTGCAACTGCGCCCAATAACATGGAATTTGCCGCGGC
CTTAGTTGCTAACCACACTGGAATCACCCAGGTGTTTAAGCGCATCTTGGGACAGTTCGATAAGCTTTGGTCCCGTAAGGCGTTCTCTCACTGGTACACC
GACTCTGGATTAACCGAAGAAGATATCGTCAACGCACGTAATGATATCGCGAAGTTGGTCGAGGAGTACGAAGAGACTCTTAAGTTCAAGAGCAAGGATA
AATCGGGAGAAGAAGTTGCGGCTGCGGAGATTGCAGAAAACAAGGGGAGCGAAAACCTTTATTTTCAATCGGGTCACCACCATCACCATCACCACCACCC
ATCTTAGTATATTAGTTAAGTATAAGAAGGAGATATACATATGTCCCGCGAAATTATCTCTATCCACGTAGGACAAGCAGGTTTACAAGTTGGAGCCGCT
TTTTGGCGCACCGTCCTTGAGGAACATGGGTTAGGAAACGACGGGAGCGTAGTTGACGCTAGTAAGGAGAAGTCTGCAGTTTATTTCAAGCAGGCTGGAA
ATAATAAGTTCGTACCACGCGCGATTTTGGTAGACCTGGAGCCCATGGTTATCAAAACAATTATGAATGATTTACCTGGGTTATTCGACCCAGCATATAC
AATTCATGGCCAGAGCGGAGCCGCTAACAACTGGGCCCGTGGGTACCGTGGTGCTGATGGCGAAGTCAACGTCGACGATGTACTGAAAATCGTAGAAAAA
GCGGTCTCTGAGACCGACTCATTGCAAGGTTTCTCAATCTCTCACTCTATCGGCGGAGGAACGGGATCTGGCTTAGGGAGTCAAATTTTAAAAAAATTGA
AAGAAACGTACCCGAAATATCCAATTATCACGTTTAGCGTCTTCCCCTCGCCACTTATTTCAGACGCAGTAACTGAACCTTATAATTCAGTTTTTGCCTT
GGACCATTTAATCGAGTACGCAGACGAGACAATTGTCTTAGATAATCACGCTTTGTATAATTTAACTAAGAATAACATGGGCATCAAATCCCCTAGCTAC
GCCGACTTAAACAAAATCATCAGTTGGGTCATGTCAGGGGTCACCGCCAGTTTGCGTTTCAAAGGTGATTTGAATACTGACCTTAAGGAGCTTTTAGTTA
ATCTTGTTCCATTTCCTCGTCAGCATTTCCTTACATCGACGTTTAGCCCAATCAAAAATGGCACTGGCAGTTTCGAGGAGGTGACCACACACGACATTAT
CAACCAATTGTTCGATCCGAATAATGCGATGTCAGACACTAACTTTGACGAAGGCGCATACATTGCGTCCGTAGTGATGATGCGTGGCCACGAGGTGAAG
ACGAATGCCGTCAATTCGTCACTGGCAGAAATTAAAGGGAAGTTACGTTTTAGTGAGTTTATCCCCACCGGCATCAAGACGGGTGTGACGTCAGAAACGC
TTGTTGGTTTCGAATCGACAGGCTTGAACTTGAGTAATCATACAGGTATCACTAAGGTCTTCAAACGCCTTGCCGCTCAGTTCGATGCGATGTACGAACG
TGACGCATTCGTGCATTGGTATCTTGGCGAAGGTATGTCTAAGGATGACATGGAGAAGGCCCGCGAGAACTTGGGTAAACTGACAACCGAATACGACACT
TCCGTGGGTTCAGAAAACTTGTACTTTCAATCCGCCTGGTCGCACCCACAGTTCGAGAAAGGAGGAGGGAGTGGTGGGGGCTCAGGTGGGTCTGCCTGGT
CCCACCCTCAATTTGAAAAATAACGATCCGGCTGCTAACAAAGCCCGAAAGGA

Figure S2B. AtubAB untagged expression construct, codon optimised for expression in *E. coli*. Blue: untagged *atubA*, red: untagged *atubB.* Sequence of the gBLOCK used to make expression plasmid pHis17-AtubAB. RBSs have been underlined.

GTTTAACTTTAAGAAGGAGATATACATATGTCTGAAGTCGTTGTTGTCGCGGTGGGCCAAGCTGGTAATCAGATTGCGGATCAGTTTTGGCAGCTTATTG
CCCAGGAACATAAGATTGGCCTGGACGGAATGCCTACGAACGGTGAGCCTACGGGGCGCACCGATGTCTTTTTTCGTAAAGTATCCAACCGTTACGTACC
CCGCGCGGTGTTGGTCGACCTTGAGCCGGCTGTGCTGAAGTCTGTCAGTGACGATGCCAAGCCGAATTTCTACGACCCGAAGAACAAAGTGCACGGGGCC
GATGGCGCCGGAAATAATTTTGCGGTGGGTCACACGGAAATGGGAGCGCAGTACTTGGAACAAGTAATTGGCAAAATCAAAAACGAGGTAGAGAAAACCG
ACTCATTGGGTGGGTTTATTGTTACACACAGTGTTGGAGGTGGTACCGGTTCTGGATTTGGAACTTTGATCATGCAGAATATCCGTAAAGAATATCCAAA
CACTCCGCTTTTGAGTTTTTCAATTTACCCCTCCCCCCGTATCTCCGATGTGCTGACCGAGCCATATAATGCGATCTTCAGTAGTTATCGCTTAGTGCGT
GACACATCATGCAGTATTATTTACGACAATGACGCATTGTATACCATCTGTCAGAACTTAGGGATTGCAGAACCAACATACTCTGACTTGAACAAGCTTA
TTTCACAAAGTATGGTCAATATCACCGCGGGTATGCGCTTTGTTGGTACCTTAAACCTTGACCTTCGCAAACTGATTACAAACTTGGTACCCCATCCGCG
TTTACACTTTCTTATGACTAGTACGGCACCGCTGGTAGAATCAGGTGGTTTCGATTCGCAATCCATTGAAAAAACGGCCGAGGTCCTGTTTGAAAAATCG
AATATCCAAGCTCAGGTGAATACGGAAGATGGCGCCTATATTTCAACAACCGTTTTGTTTCGCGGGAACGTCAACCCAGCTGACATGGATACTGCTCTTC
GTGCAATGAAGCAAAAGCTGAAATTTGTCTCGTTTATTCCGACCGGTTTCAAGACAGGACTGTCTGCAACTGCGCCCAATAACATGGAATTTGCCGCGGC
CTTAGTTGCTAACCACACTGGAATCACCCAGGTGTTTAAGCGCATCTTGGGACAGTTCGATAAGCTTTGGTCCCGTAAGGCGTTCTCTCACTGGTACACC
GACTCTGGATTAACCGAAGAAGATATCGTCAACGCACGTAATGATATCGCGAAGTTGGTCGAGGAGTACGAAGAGACTCTTAAGTTCAAGAGCAAGGATA
AATCGGGAGAAGAAGTTGCGGCTGCGGAGATTGCAGAAAACAAGTAACCATCTTAGTATATTAGTTAAGTATAAGAAGGAGATATACATATGTCCCGCGA
AATTATCTCTATCCACGTAGGACAAGCAGGTTTACAAGTTGGAGCCGCTTTTTGGCGCACCGTCCTTGAGGAACATGGGTTAGGAAACGACGGGAGCGTA
GTTGACGCTAGTAAGGAGAAGTCTGCAGTTTATTTCAAGCAGGCTGGAAATAATAAGTTCGTACCACGCGCGATTTTGGTAGACCTGGAGCCCATGGTTA
TCAAAACAATTATGAATGATTTACCTGGGTTATTCGACCCAGCATATACAATTCATGGCCAGAGCGGAGCCGCTAACAACTGGGCCCGTGGGTACCGTGG
TGCTGATGGCGAAGTCAACGTCGACGATGTACTGAAAATCGTAGAAAAAGCGGTCTCTGAGACCGACTCATTGCAAGGTTTCTCAATCTCTCACTCTATC
GGCGGAGGAACGGGATCTGGCTTAGGGAGTCAAATTTTAAAAAAATTGAAAGAAACGTACCCGAAATATCCAATTATCACGTTTAGCGTCTTCCCCTCGC
CACTTATTTCAGACGCAGTAACTGAACCTTATAATTCAGTTTTTGCCTTGGACCATTTAATCGAGTACGCAGACGAGACAATTGTCTTAGATAATCACGC
TTTGTATAATTTAACTAAGAATAACATGGGCATCAAATCCCCTAGCTACGCCGACTTAAACAAAATCATCAGTTGGGTCATGTCAGGGGTCACCGCCAGT
TTGCGTTTCAAAGGTGATTTGAATACTGACCTTAAGGAGCTTTTAGTTAATCTTGTTCCATTTCCTCGTCAGCATTTCCTTACATCGACGTTTAGCCCAA
TCAAAAATGGCACTGGCAGTTTCGAGGAGGTGACCACACACGACATTATCAACCAATTGTTCGATCCGAATAATGCGATGTCAGACACTAACTTTGACGA
AGGCGCATACATTGCGTCCGTAGTGATGATGCGTGGCCACGAGGTGAAGACGAATGCCGTCAATTCGTCACTGGCAGAAATTAAAGGGAAGTTACGTTTT
AGTGAGTTTATCCCCACCGGCATCAAGACGGGTGTGACGTCAGAAACGCTTGTTGGTTTCGAATCGACAGGCTTGAACTTGAGTAATCATACAGGTATCA
CTAAGGTCTTCAAACGCCTTGCCGCTCAGTTCGATGCGATGTACGAACGTGACGCATTCGTGCATTGGTATCTTGGCGAAGGTATGTCTAAGGATGACAT
GGAGAAGGCCCGCGAGAACTTGGGTAAACTGACAACCGAATACGACACTTCCGTGTAACGATCCGGCTGCTAACAAAGCCCGAAAGGA

Figure S2C. Tagged AtubAhBs 360 nm 90° light scattering. Experiment the same as in Figure 2F but using tagged AtubAhBs protein instead (blue trace), which appears in this assay to polymerises more and depolymerises faster than untagged AtubAB (red trace).

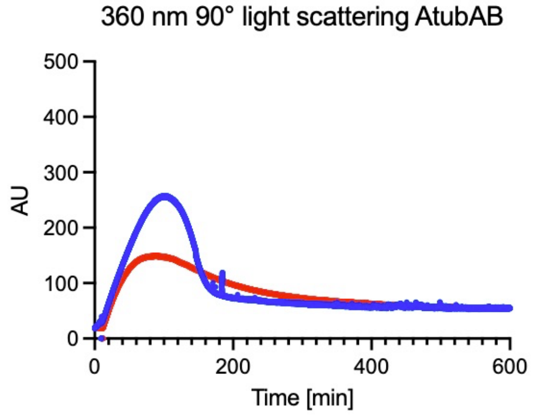

Figure S2D. AtubAB pelleting assays with tubulin-directed small-molecule compounds. A panel of tubulin-directed drugs and inhibitors were tested against AtubAB polymerisation in a pelleting assay. Maytansine (MAY), a tubulin polymerisation inhibitor effectively inhibits pelleting of AtubAB in this assay. The same effect is shown in different experiments in Figure 2G and 2H. Key: KT (Kari AtubAB, nothing-added control), DMSO (dimethyl sulfoxide control), LAU (laulimalide), PTX (paclitaxel), ZAM (zampanolide), DIS (discodermolide), EPO (epothilone), MAY (maytansine), PIR (pironetin), GAT (gatorbulin), VIN (vinblastine), COL (colchicine), POD (podophyllotoxin), SAB (sabizabulin), NOC (nocodazole) and PLI (plinabulin). S/N, supernatant.

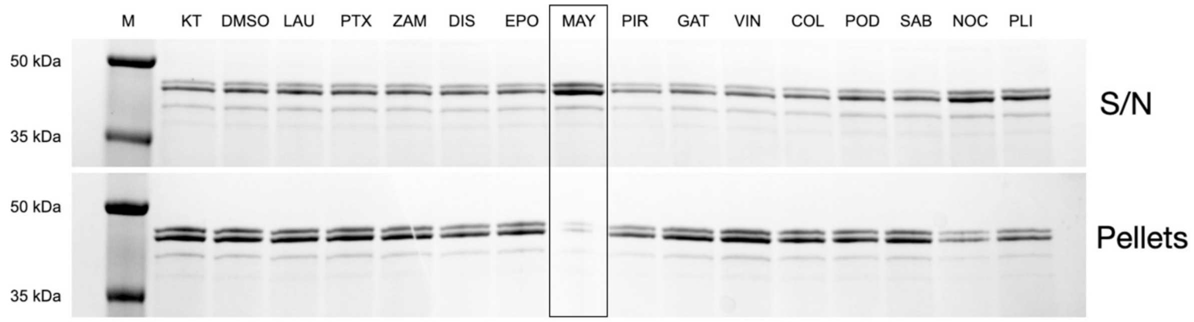

Figure S2E. Affinity of maytansine for AtubAB. i) Binding affinity measurement using the fluorescently labelled maytansine compound FcMaytansine (Methods). The affinity was determined to be K_d= 87 ± 17 nM (mean ± SEM, *n* = 4, see Methods). ii) Competition experiment to determine the AtubAB affinity of unlabelled maytansine. K_d = 5.1 ± 0.2 μM.

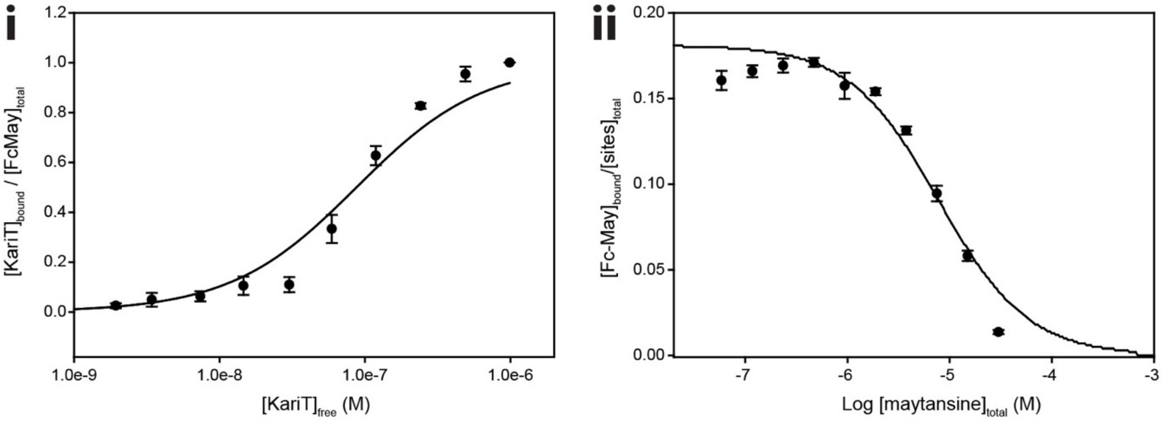

Figure S2F. Superposition of tubulin complexed with maytansine and AtubAB AlphaFold 3 model. β tubulin:maytansine complex (PDB 4TV8) (*30*) superimposed on AtubA. The binding pocket is highly conserved - all interacting residues are identical except β tubulin(K105)/AtubA(V100) and β tubulin(V181)/AtubA(L176).

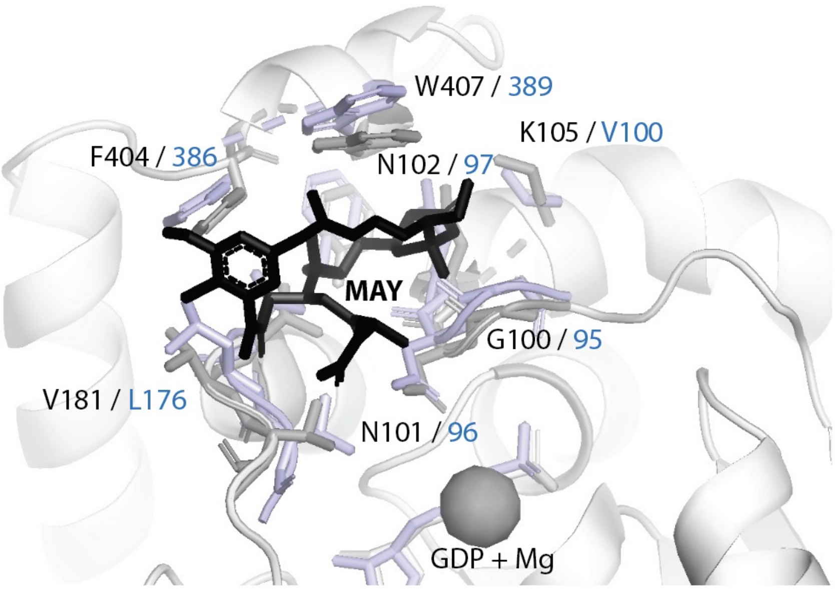

Figure S3A. Distribution of lamellae thicknesses after focussed ion beam (FIB)-milling of *E. coli* C41(DE3) cells over expressing untagged AtubAB. The lamellae were used to perform electron cryotomography with subsequent subtomogram averaging to solve the *in situ* structure of AtubAB (Figures 3B, S3D). Thicknesses were determined with the software GeoLlama after tomography (*69*). Note that 50 lamellae were prepared but three were discarded before reconstructing them (47 in total, Supplementary Table 1).

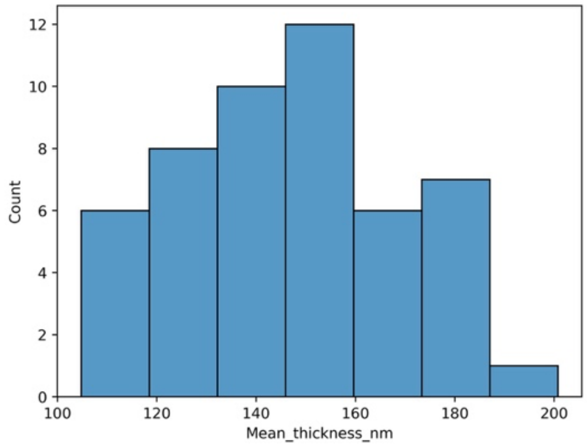

Figure S3B. Cryo-ET / subtomogram averaging (STA) processing scheme. *E. coli* C41(DE3) cells over expressing untagged AtubAB and FIB-milled.

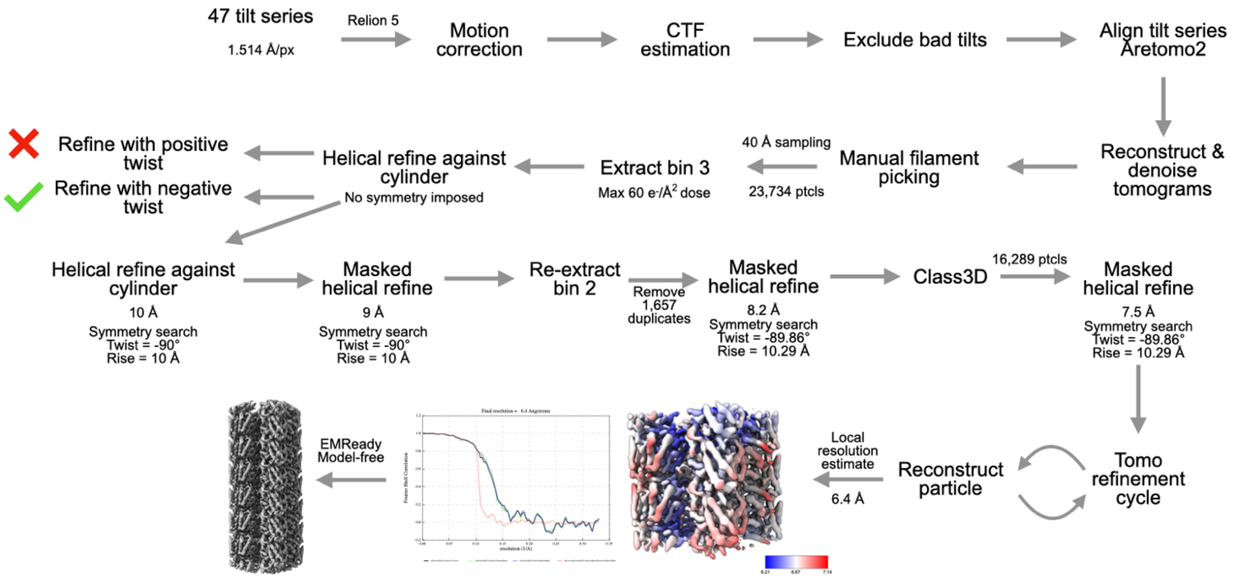

Figure S3C. Low-magnification negative staining of AtubAB. Polymerisation performed in BRB80 + 500 mM potassium glutamate Polymerisation Buffer with GDP, GTP and GMPCPP nucleotides (scale bars = 5 μm). GTP and GMPCPP-containing reactions show many filaments. See also the corresponding pelleting experiment in Figure 2E.

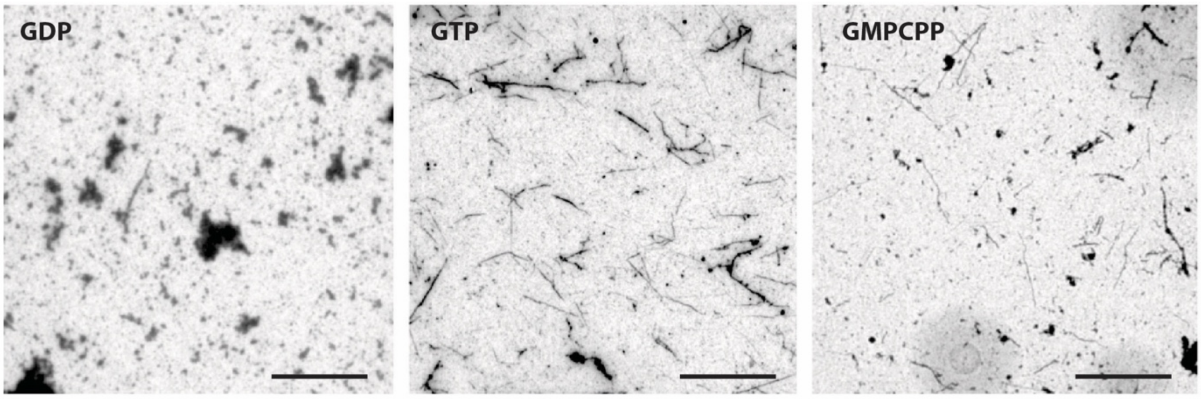

Figure S3D. Cryo-EM processing scheme: helical processing and symmetry breaking of AtubAB mini microtubules.

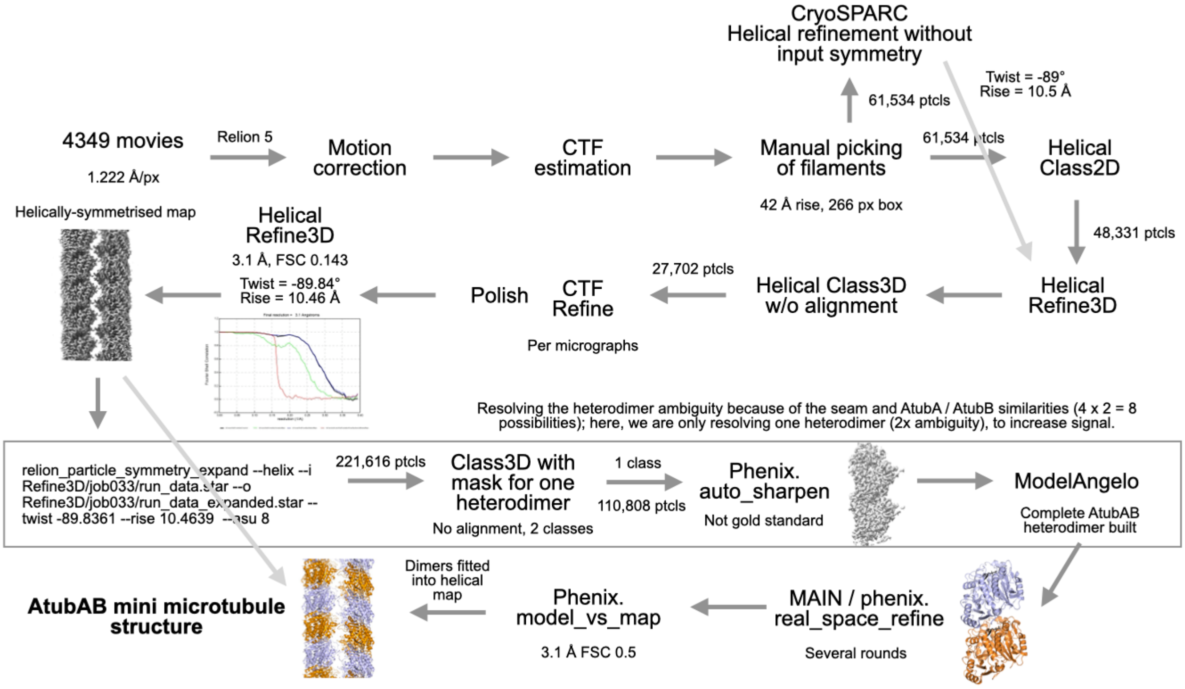

Figure S3E. AtubAB model-free and subtomogram-averaged *in situ* map (Figure 3B), fitted with the *in vitro* cryo-EM atomic model (Figure 3F). Note that the colours are different here because A and B could not be identified in the 6.4 Å resolution map. Helices are resolved; sheets are not, in line with the resolution estimate.

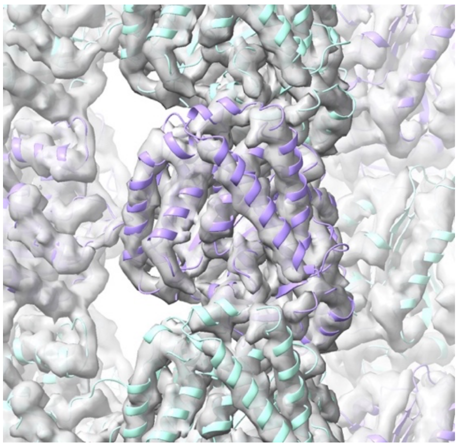

Figure S3F. Superposition of AtubAB mini microtubule heterodimer, as determined in this study, with eukaryotic αβ tubulin in a microtubule. i) AtubA (top, green) superimposed on PDB 7QUP (*Drosophila melanogaster* microtubule, grey) β tubulin (2251 out of 2650 residues superimposed in PyMOL, RMSD = 0.897 Å) (*6*); right: AtubB (bottom) superimposed on PDB 7QUP α tubulin (2227 out of 2653 residues superimposed in PyMOL, RMSD = 0.918 Å). 7QUP is grey, AtubAB is green. See also Movie M2. **ii)** Same superposition as in i (right, AtubB on α tubulin), highlighting similarities in the nucleotide binding pockets. **iii)** The M-loops in AtubAB and eukaryotic microtubules are similar but support dramatically different inter-protofilaments angles as needed because of the different protofilaments numbers (13 versus 4 for AtubAB).

i

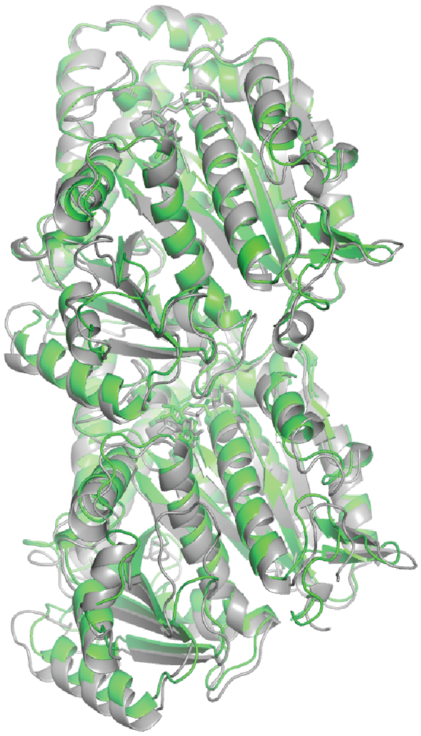

AtubA aligned against PDB 7QUP β tubulin (top subunits, tubulin grey), RMSD = 0.897 Å

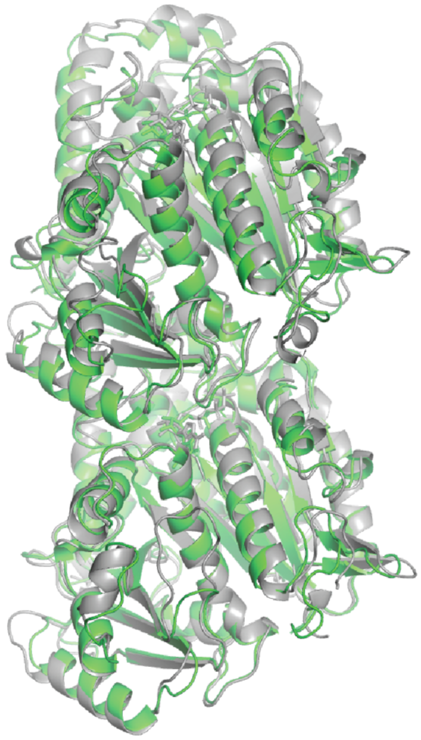

AtubB aligned against PDB 7QUP α tubulin (bottom subunits), RMSD = 0.918 Å

ii

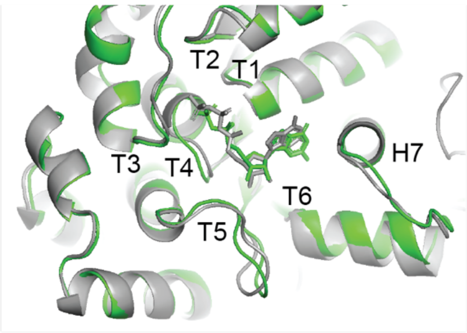

AtubB aligned against PDB 7QUP α tubulin: nucleotide binding pockets. AtubB is bound by GDP, tubulin by GTP.

iii

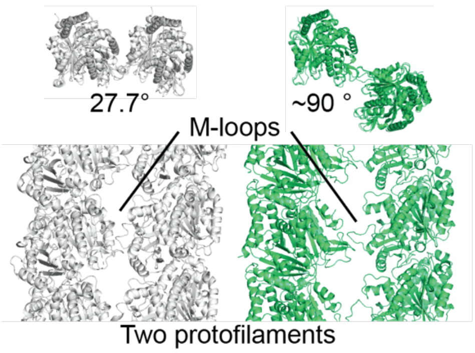

M-loops hold microtubule protofilaments together. In 13-pf and 4-pf microtubules they support dramatically different inter-pf angles.

Figure S3G. Purified AtubAB mostly contains GDP. The purified untagged AtubAB protein was extracted for nucleotides and using HPLC they were compared against GDP/GTP and guanosine standards (left). The sample on the right shows that purified AtubAB contains very little GTP, meaning that the stable AtubAB heterodimer as purified here (using harsh conditions) does most likely trap a GDP in AtubB, which is in line with the finding that the polymerised protein filaments also contained only GDP (Figure 3K). Numbers refer to elution time in min.

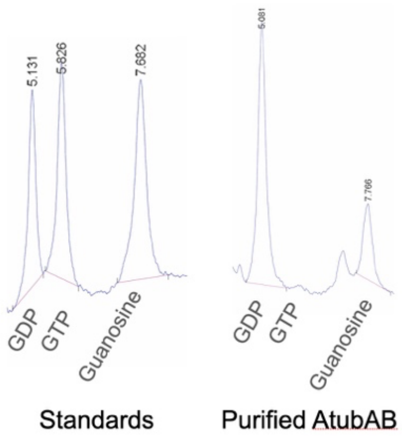

Figure S3H. AtubAB cryo-EM for single-particle analysis (SPA). i) Cryo-EM image of untagged AtubAB for single particle analysis (SPA; no nucleotide added, structure Figure 3K, right). Bandpass filtered for clarity. Scale bar 100 nm. ii) 2D class averages after picking particles, revealing top and side views of the AtubAB dimers. Scale bar 10 nm.

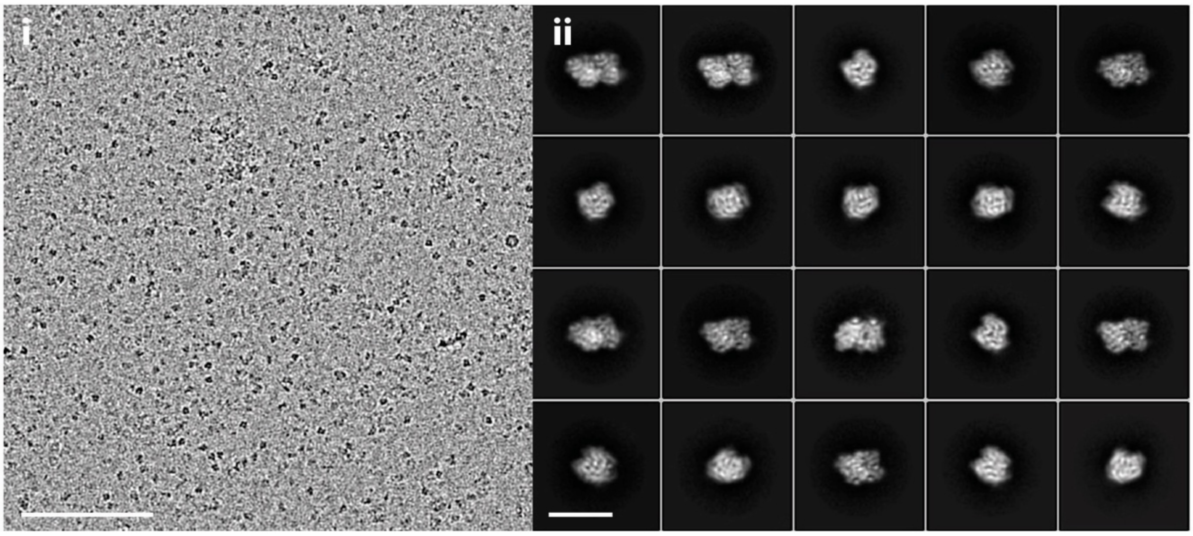

Figure S3I. Cryo-EM single-particle processing scheme of untagged AtubAB un-polymerised heterodimer (SPA).

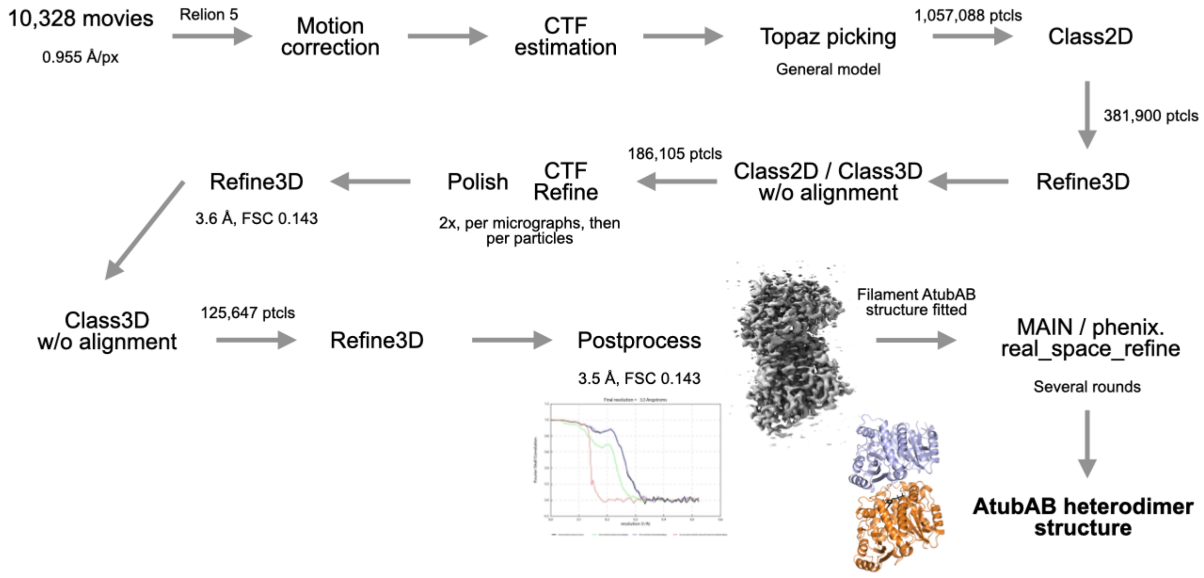

Figure S4A. TIRF microscopy of AtubAB filament dynamics. TIRF microscopy, AtubAB polymerisation and kymographs as in Figure 4A-C. Red: GMPCPP-stabilised seeds (not depolymerising), cyan: AtubAB with GTP/GDP, dynamic**. i)** Field of view using untagged proteins, only (4 µM final concentration). Scale bar 5 µm. **ii) and iii)** Further examples of dynamic untagged AtubAB filaments (4 µM final concentration), similar to Figure 4C, showing slow growth at the minus ends (left) and fast growth and dynamic instabilities at the plus ends (right). **iv)** Same as ii and iii for a sample with 20% tagged, unlabelled AtubAhBs added (Methods), to increase turnover at higher concentrations needed to get highly populated field views (as in Figure 4A) (5 µM, see Methods). Scale bars = 2 min and 2 µm.

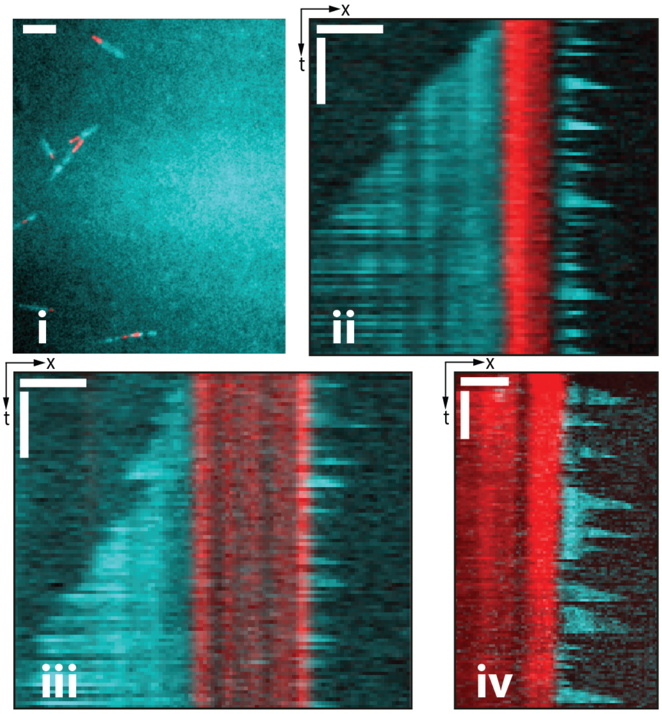

Figure S4B. Quantifications of AtubAB mini microtubule dynamics as observed by TIRF microscopy. Relate to Figures 4C and S4A. **i) & ii)** AtubAB GMPCPP seeds were polymerised and filament growth rates **(i)** (minus ends, median speed of growth 0.48 µm/min; plus ends, median speed of growth 1.80 µm/min) and maximal lengths before undergoing catastrophe **(ii)** were measured. p values from two-sided Kruskal-Wallis tests for non-parametric samples are indicated; n, number of individual growth events quantified from at least three independent experiments. Thick lines, median; thin lines, quartile. **iii)** Cumulative AtubAB filament lifetime distributions of mini microtubules grown at the minus (blue) and plus ends (green). Mean lifetime estimates +/- error (lifetime at half cumulative distribution): 451.9 +/- 55.5 s (minus ends) and 45.1 +/- 1.6 s (plus ends). Line, gamma distribution fits.

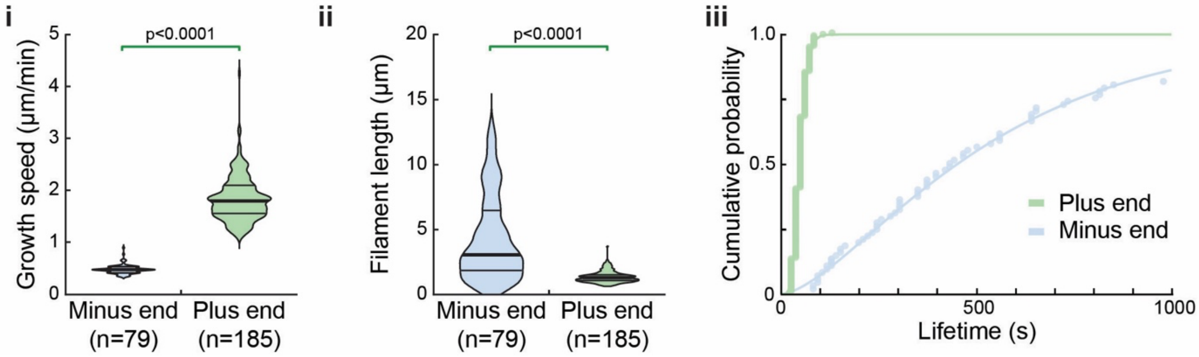

Supplementary Figure S5. Phylogeny reconstructed using IQ-TREE 3 under the model Q.pfam+C50+G4+PMSF of an alignment including artubulin and a CetZ-related Asgard archaeal clade as outgroup, aligned with MAFFT-linsi, trimmed with trimAl of sites containing over 50 % gaps, pruned of sequences formed by over 50 % gaps, containing 488 sequences and 431 columns. Branch support values represent Transfer Bootstrap Expectation. Branch length legend indicates substitutions per site. The same tree, indicating Felsenstein Bootstrap Proportions is shown in Supplementary Figure S11.

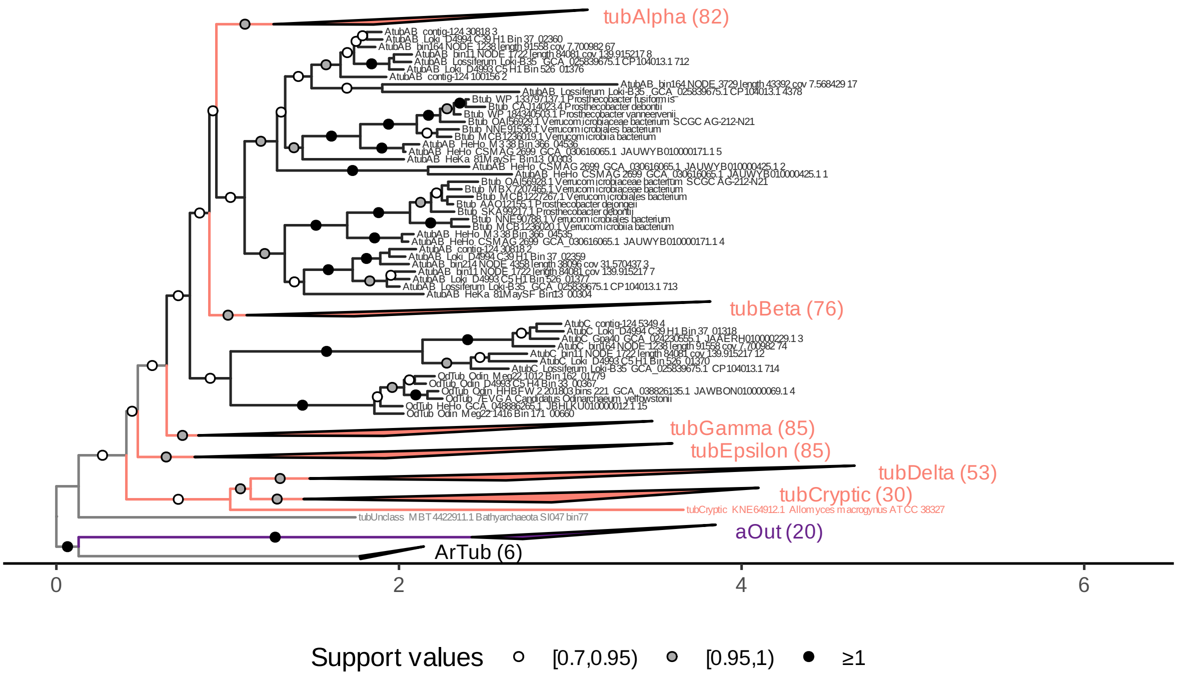

Supplementary Figure S6. Phylogeny reconstructed using IQ-TREE 3 under the model Q.pfam+C50+G4+PMSF of an alignment including CetZ and a CetZ-related Asgard archaeal clade as outgroup, aligned with MAFFT-linsi, trimmed with trimAl of sites containing over 50 % gaps, pruned of sequences formed by over 50 % gaps, containing 1590 sequences and 337 columns. Branch support values represent Transfer Bootstrap Expectation. Branch length legend indicates substitutions per site. The full tree, indicating Felsenstein Bootstrap Proportions is shown in Supplementary Figure S12.

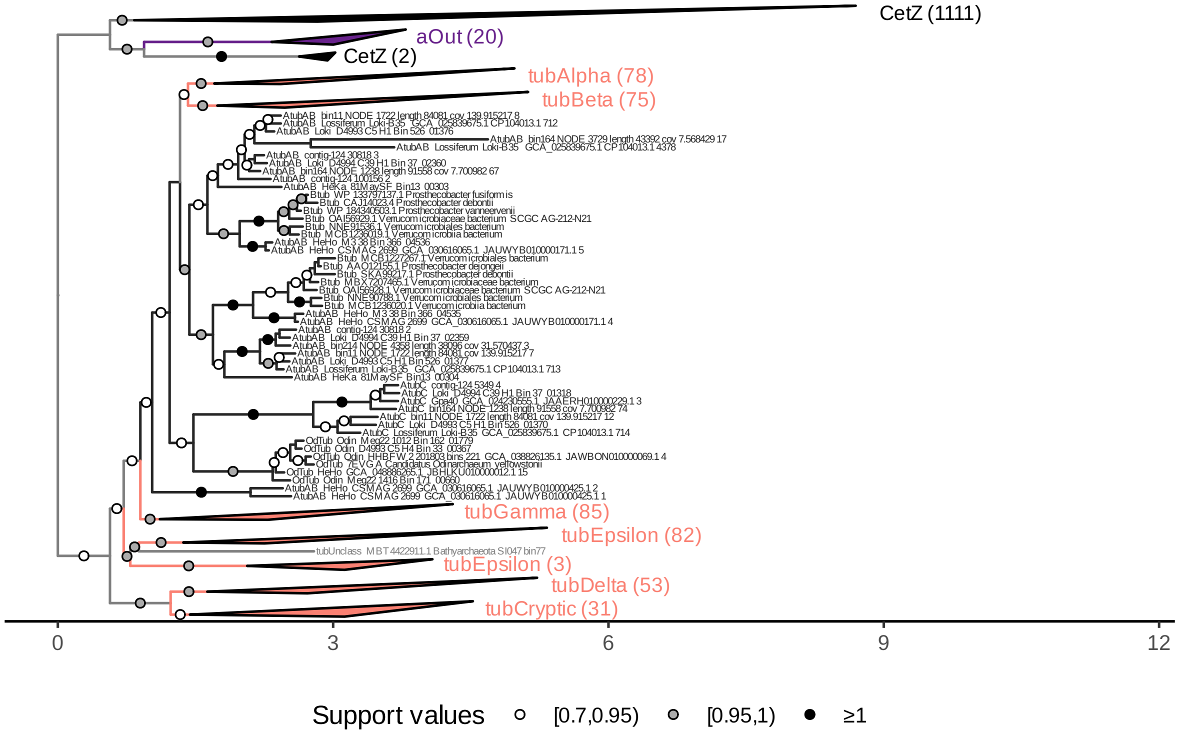

Supplementary Figure S7. Phylogeny reconstructed using IQ-TREE 3 under the model Q.pfam+C50+G4+PMSF of an alignment including artubulin and a CetZ-related Asgard archaeal clade as outgroup, aligned with MAFFT-linsi, trimmed with trimAl of sites containing over 90 % gaps, pruned of sequences formed by over 50 % gaps, and reconstructed while trimming the 10 % of sites with the lowest likelihood scores during the reconstruction with IQ-TREE 3. Tree used as input to IQ-TREE 3 contained 477 sequences and 578 columns. Branch support values represent Transfer Bootstrap Expectation. Branch length legend indicates substitutions per site. The full tree, indicating Felsenstein Bootstrap Proportions is shown in Supplementary Figure S13.

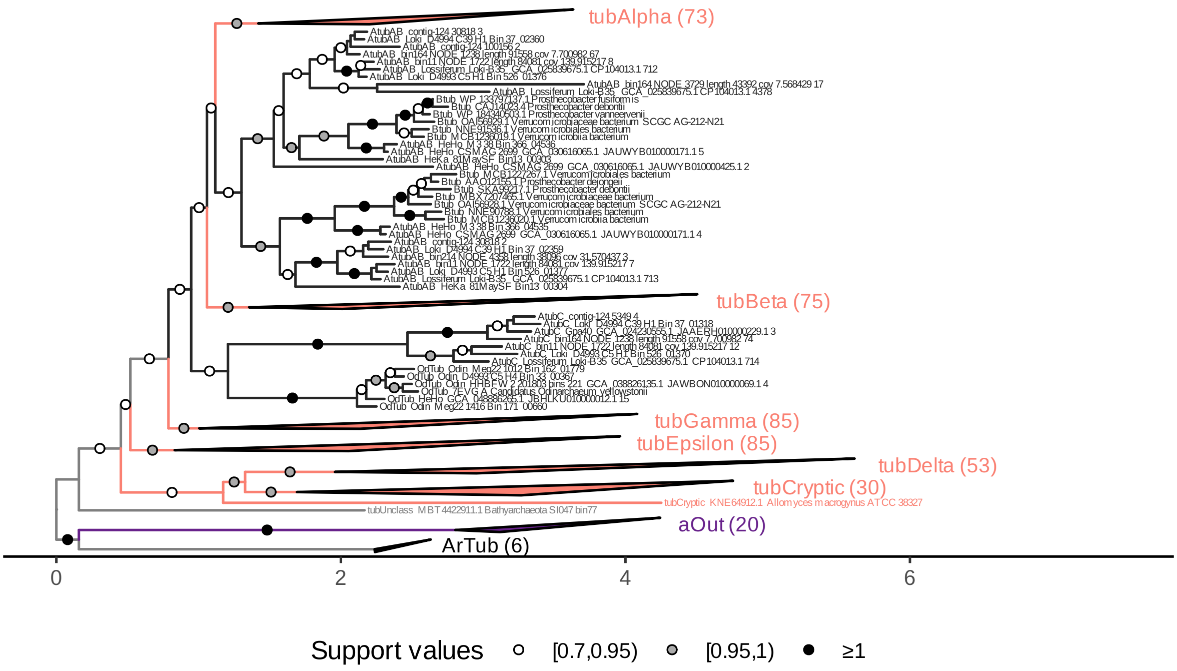

Supplementary Figure S8. Phylogeny reconstructed using IQ-TREE 3 under the model Q.pfam+C50+G4+PMSF of an alignment including artubulin as outgroup, aligned with MAFFT-linsi, trimmed with trimAl of sites containing over 50 % gaps, pruned of sequences formed by over 50 % gaps, and reconstructed while trimming the 10 % of sites with the lowest likelihood scores during the reconstruction with IQ-TREE 3. Tree used as input to IQ-TREE 3 contained 299 sequences and 435 columns. Branch support values represent Transfer Bootstrap Expectation. Branch length legend indicates substitutions per site. The full tree, indicating Felsenstein Bootstrap Proportions is shown in Supplementary Figure S14.

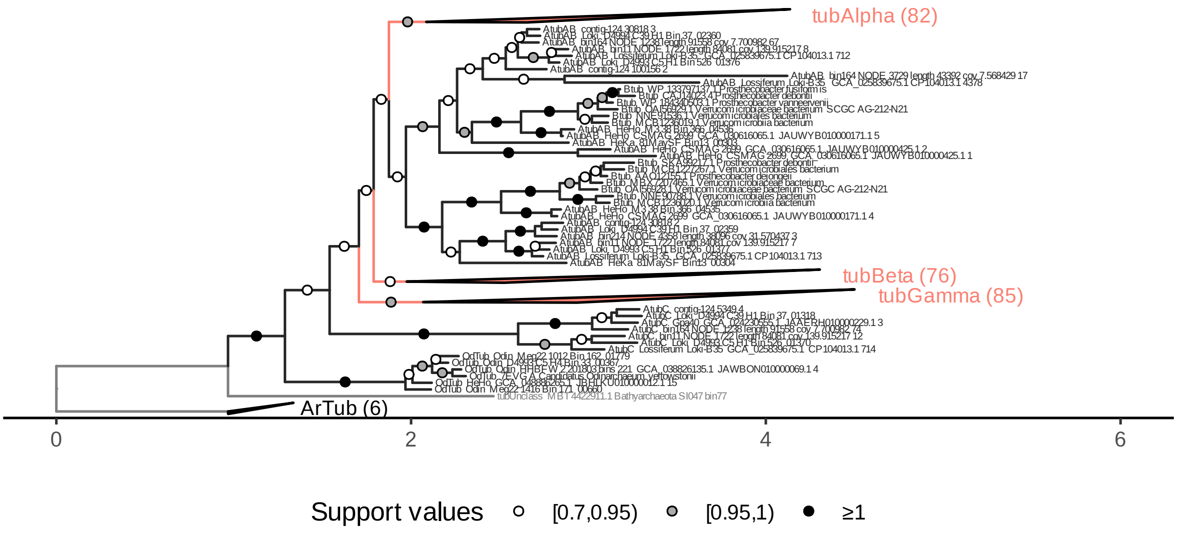

Supplementary Figure S9. Phylogeny reconstructed using IQ-TREE 3 under the model Q.pfam+C50+G4+PMSF of an alignment including artubulin and CetZ as outgroup, aligned with MAFFT-linsi, trimmed with trimAl of sites containing over 50 % gaps, pruned of sequences formed by over 50 % gaps, containing 1406 sequences and 339 columns. Branch support values represent Transfer Bootstrap Expectation. Branch length legend indicates substitutions per site. The full tree, indicating Felsenstein Bootstrap Proportions is shown in Supplementary Figure S15.

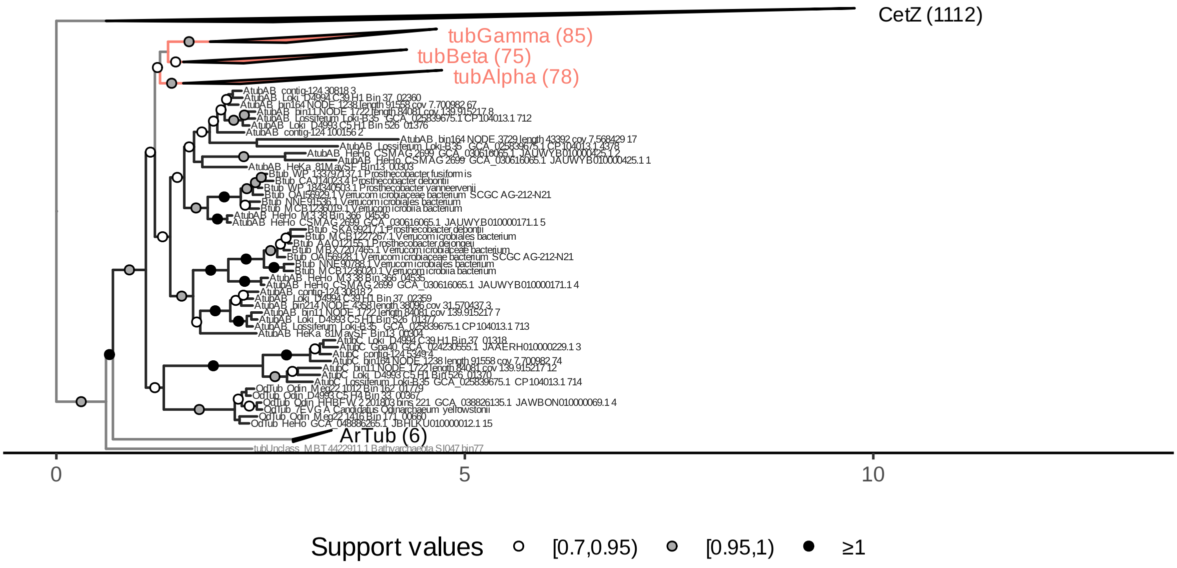

Supplementary Figure S10. Phylogeny reconstructed using IQ-TREE 3 under the model Q.pfam+C50+G4+PMSF of an alignment including CetZ as outgroup, aligned with MAFFT-linsi, trimmed with trimAl of sites containing over 50 % gaps, pruned of sequences formed by over 50 % gaps, and reconstructed while trimming the 10 % of sites with the lowest likelihood scores during the reconstruction with IQ-TREE 3. Tree used as input to IQ-TREE 3 contained 1400 sequences and 337 columns. Branch support values represent Transfer Bootstrap Expectation. Branch length legend indicates substitutions per site. The full tree, indicating Felsenstein Bootstrap Proportions is shown in Supplementary Figure S16.

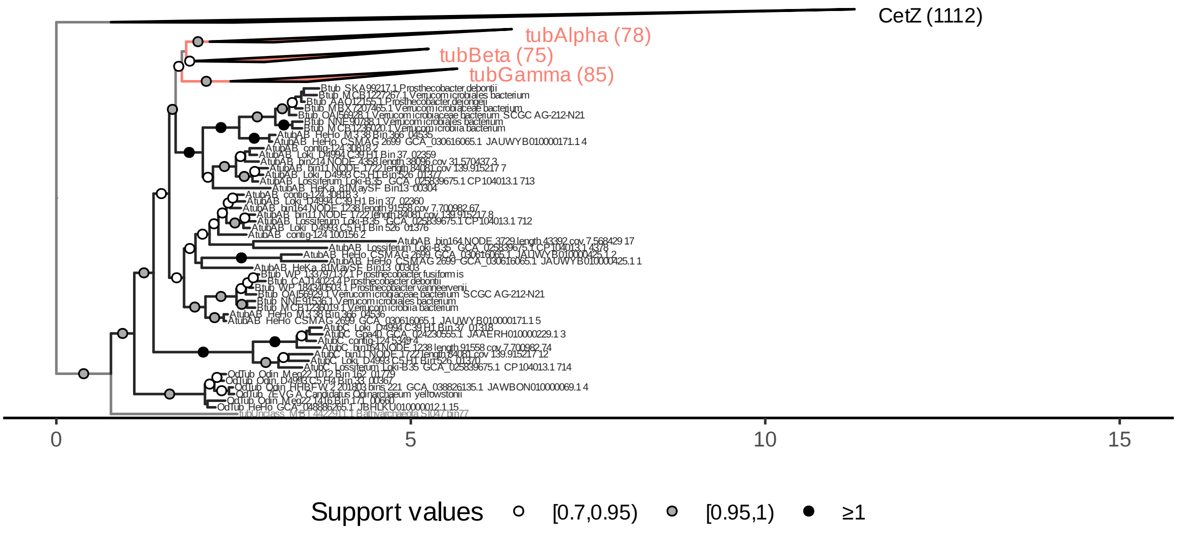

Supplementary Figure S11. Full phylogeny corresponding to Supplementary Figure S5, indicating Felsenstein Bootstrap Proportions as branch support values.

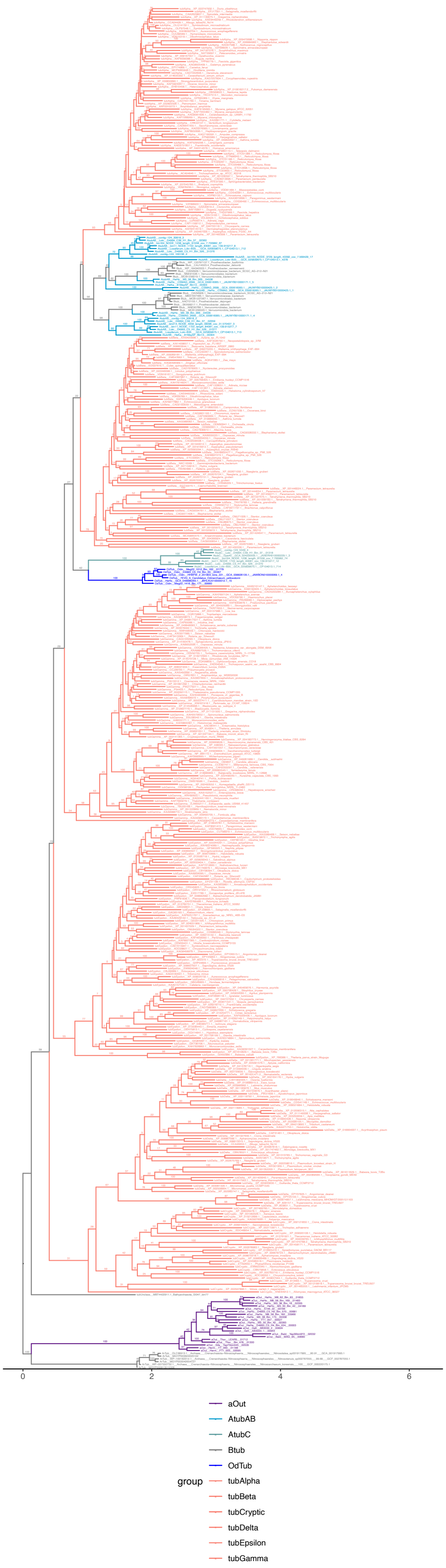

Supplementary Figure S12. Full phylogeny corresponding to Supplementary Figure S6, indicating Felsenstein Bootstrap Proportions as branch support values.

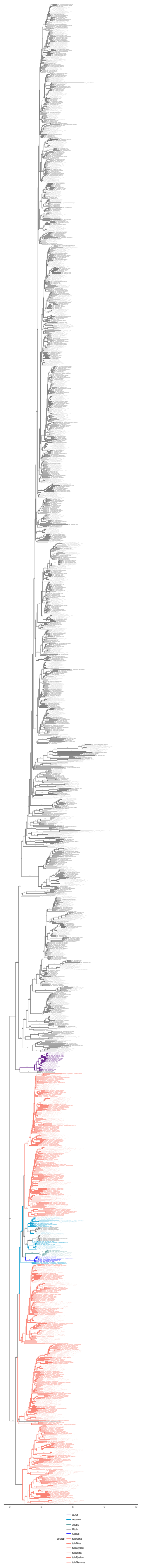

Supplementary Figure S13. Full phylogeny corresponding to Supplementary Figure S7, indicating Felsenstein Bootstrap Proportions as branch support values.

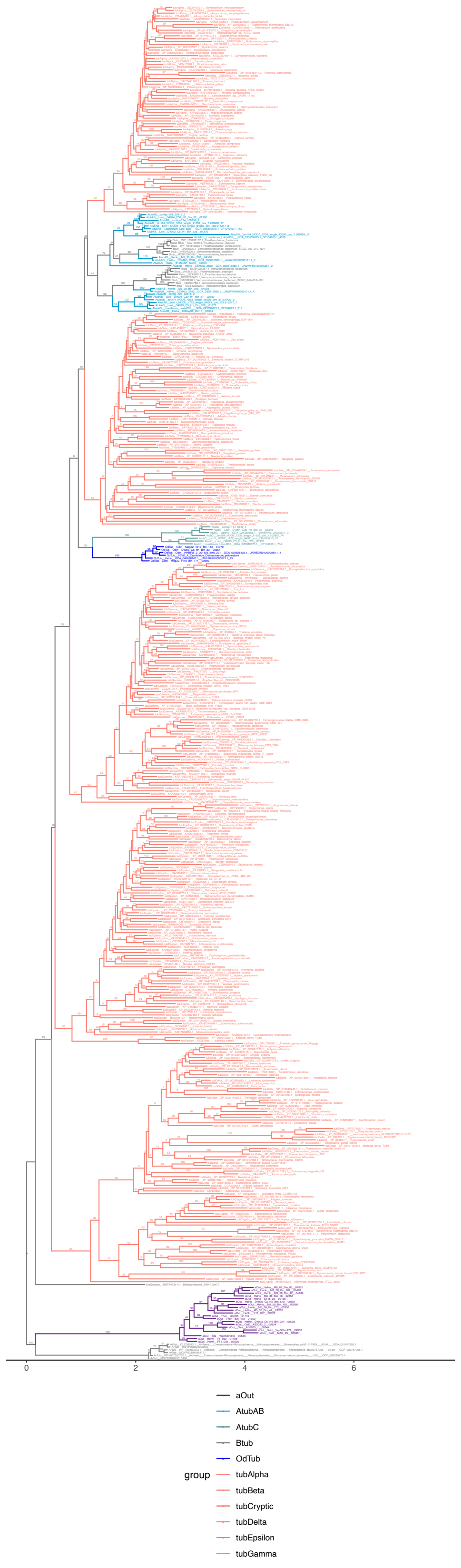

Supplementary Figure S14. Full phylogeny corresponding to Supplementary Figure S8, indicating Felsenstein Bootstrap Proportions as branch support values.

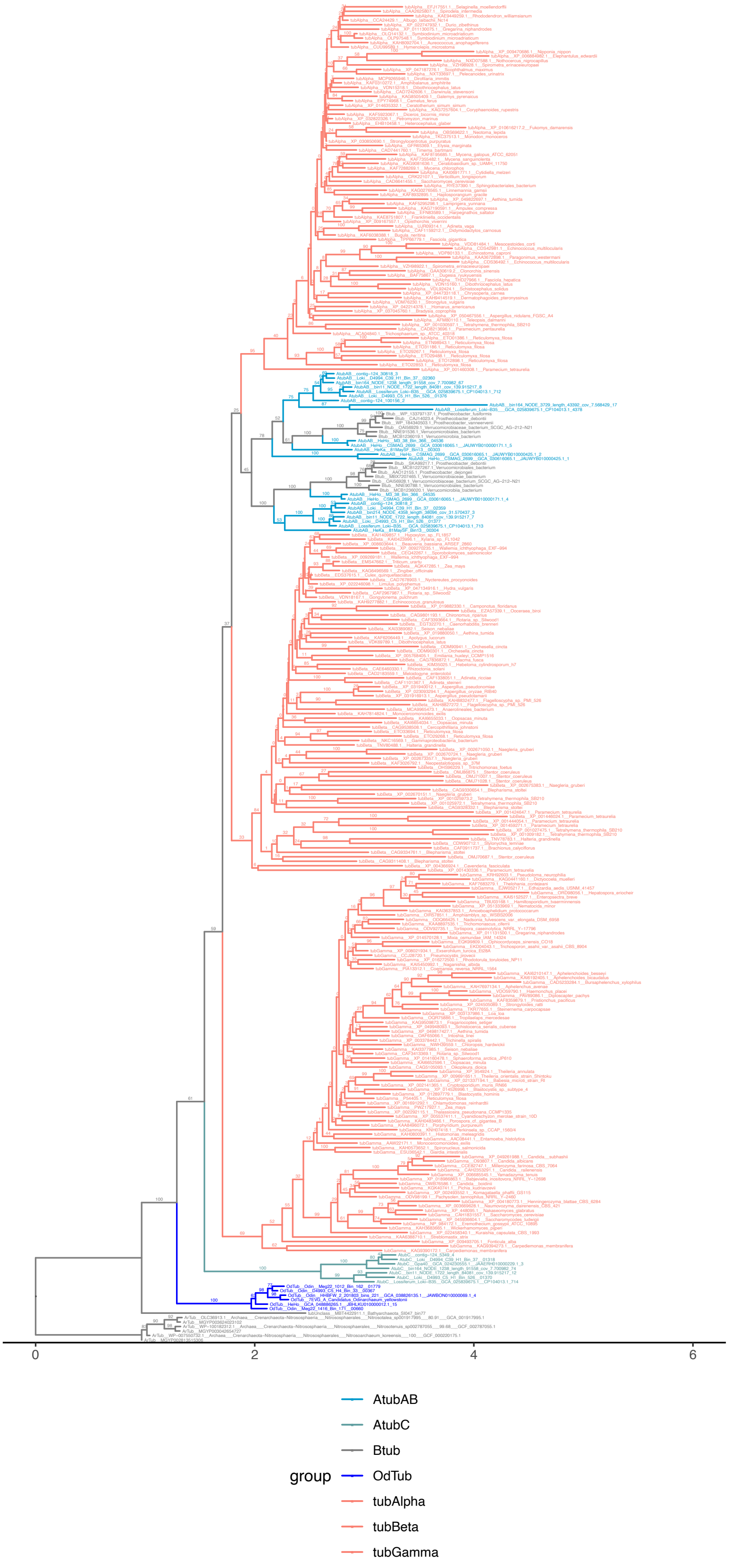

Supplementary Figure S15. Full phylogeny corresponding to Supplementary Figure S9, indicating Felsenstein Bootstrap Proportions as branch support values.

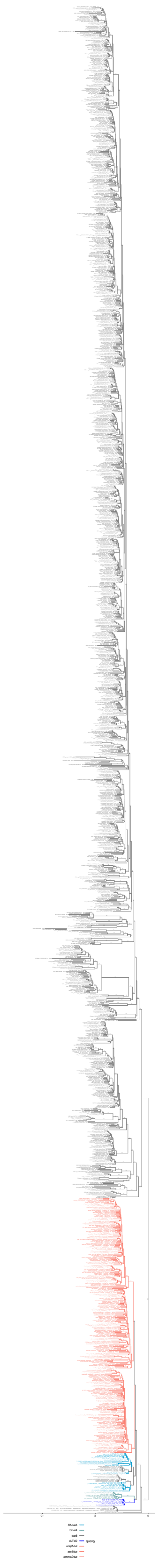

Supplementary Figure S16. Full phylogeny corresponding to Supplementary Figure S10, indicating Felsenstein Bootstrap Proportions as branch support values.

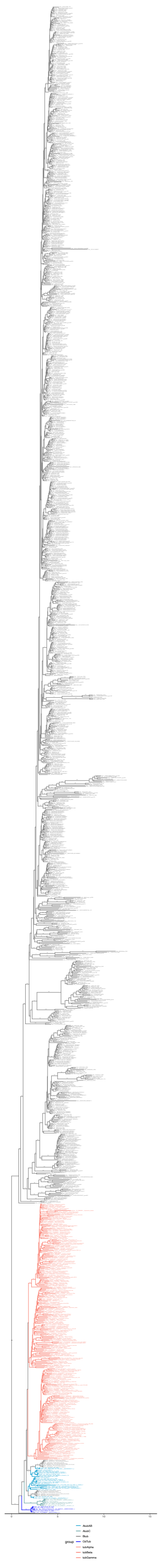

SUPPLEMENTARY TABLE

Supplementary Table S1. Cryo-EM/ET data, refinement and validation statistics.

	AtubAB mini microtubule in *E. coli* cells by cryo-ET with subtomogram averaging; EMD-56154	AtubAB mini microtubule by cryo-EM; EMD-56153; PDB ID 9TQK	AtubAB free heterodimer by cryo-EM; EMD-56152; PDB ID 9TQJ
Proteins	Untagged AtubAB: GenBank MDH5401500.1 (M1 replaced with MSEVVVV); MDH5401501.1	Untagged AtubAB: GenBank MDH5401500.1 (M1 replaced with MSEVVVV); MDH5401501.1	Untagged AtubAB: GenBank MDH5401500.1 (M1 replaced with MSEVVVV); MDH5401501.1
**Data and processing**			
Magnification	83,000	105,000x	130,000x
Voltage (kV)	300	300	300
Electron exposure (e–/Å^2)	152 e-/Å^2, 37 tilts at 4.1 e-/Å^2	60	50
Defocus range (µm)	-1.5 µm to -3.5 µm	-1.5 to -2.5	-1.5 to -2.5
Pixel size (Å)	1.514	1.222	0.955
Symmetry imposed	Twist = -89.824°, rise = 10.248 Å	Twist= -89.836°, rise = 10.460 Å	None
Initial particle images (no.)	47 tomograms, 44 used, 23,734 stacks	61,534	1,057,088
Final particle images (no.)	15,362 stacks	27,702	125,647
Map resolution (Å)	6.4	3.1	3.5
FSC threshold	0.143	0.143	0.143
**Refinement**			
Initial model used (PDB code)	None	*De novo* (Relion 5 - ModelAngelo)	AlphaFold3 model
Map sharpening *B* factor (Å^2)	-200	-110	-139
Model composition	Not applicable	AtubAB (heterodimer)	AtubAB heterodimer
Non-hydrogen atoms		6543	6016
Protein residues		A: 418; B: 422	A: 354; B: 420
Ligands		2 GDP (in A and B)	1 GDP in AtubB
B factors protein (Å^2)		66.4	72.8
RMSD bond lengths (Å)		0.003	0.007
RMSD bond angles (°)		0.618	1.144
Validation			
MolProbity score		1.4 (97th percentile)	1.5 (96th percentile)
Clashscore		6.55	8.77
Poor rotamers (%)		0.7	0.3
Rama Favoured (%)		97.7	98.2
Rama Disallowed (%)		0.0	0.0

SUPPLEMENTARY MOVIES

Movie S1. Electron cryotomogram (cryo-ET) of FIB-milled lamella of *E. coli* C41(DE3) cell over-expressing untagged AtubAB, showing bundles of mini microtubules crossing a cell division site (same data as shown in Figure 3A, cells expressing AtubAB as shown in Figure 2A, left).

Movie S2. Superposition of AtubAB filament dimer structure and eukaryotic (*D. melanogaster*) α/β tubulin in microtubule (PDB 7QUP). 7QUP is grey, AtubAB is green. Same as Supplementary Figure S3F, right.

Movie S3. Overview of the architecture of AtubAB four-stranded mini microtubules. Same structure as in Figure 3F.

Movie S4. AtubAB's cytomotive switch, the polymerisation-dependent conformational switch enabling filament dynamics. Morph between AtubB in the filament and AtubB in the un-polymerised dimer conformations (same as Figure 3L).

Movie S5. TIRF microscopy of AtubAB filaments revealing dynamic instability. Red: stable GMPCPP seeds. Cyan: dynamic AtubAB (GTP/GDP). The kymographs on the right show two different filaments from the overview on the left. They grow slowly at the minus ends (left side of the red GMPCPP seeds) and grow fast at the plus ends (right side), where they also undergo frequent catastrophes to display dynamic instability (very rapid depolymerisation/shortening). Some data the same as in Figures 4B & C. Scale bar 5 µm.

SUPPLEMENTARY DATA

Sequences, alignments and phylogenetic trees underlying phylogenetic analyses are at the following Figshare repository: https://doi.org/10.6084/m9.figshare.32204856.

SUPPLEMENTARY DISCUSSION

Phylogenetic analyses

Reconstructing reliable phylogenetic trees from tubulin protein sequences is particularly challenging because the alignments contain limited phylogenetic signal. Tubulins are highly conserved, (relatively) short proteins, resulting in low effective sequence variation relative to the number of taxa analysed. This low signal amplifies the impact of stochastic errors, model misspecification, and alignment uncertainty on inferred topologies. Consequently, deep or rapid divergence events are especially difficult to resolve, and alternative tree topologies often receive comparable statistical support. In this case, this translates to multiple clades robustly inferred as monophyletic (eukaryotic tubulin α, β, γ, δ, ε and cryptic clades, AtubA + BtubA, AtubB + BtubB, AtubC, and Odin tubulin), but with unclear relatedness patterns between them.

To identify recurrent topological patterns that may illuminate the evolution of tubulin sequences, we performed additional phylogenetic reconstructions varying sequence sampling. To assess possible effects of long-branch attraction due to long-branching outgroup sequences, we performed phylogenetic analyses including only the ingroup (that is, eukaryotic and prokaryotic tubulins only), or including combinations of three tubulin homologues: artubulin, CetZ, and a small group of Asgard archaeal sequences that may be associated to CetZ.

The results from these phylogenies corroborated the main findings in Figure 1A, including: 1) BtubA and BtubB clustering within AtubA and AtubB; 2) AtubA and AtubB clustering with eukaryotic α tubulin or at the base of the α and β tubulin clade; and 3) AtubC clustering with Odin tubulin (Supplementary Figures S5 & S6, below). While the affiliation of AtubC and Odin tubulin in the tubulin clade was not always clear, they tended to branch as part of a monophyletic group with α and β tubulins, AtubA-BtubA, AtubB-BtubB, and, sometimes, γ tubulin. This result remained consistent after trimming the sites with the lowest decile of low-likelihood scores (option "--robust-phy 0.9" in IQ-Tree v3.0.1) (Supplementary Figure S7) (*53*).

Notably, as explained above, the obtained results lack sufficient phylogenetic signal to produce robust branch support values. For this reason, we employed the Transfer Bootstrap Expectation (TBE) metric (*54*), which considers the average number of sequences that are 'transferred' across the bipartition in bootstrap trees, compared to the number of leaves at the light side of the bipartition (i.e. a TBE value of 0.9 indicates that 10% of the number of leaves in the light side of the bipartition are transferred on average). Thus, when applied to clades including groups with very few members, high support values need to be considered with a high degree of caution.

Given the very long branches present in the tubulin ε, δ and cryptic clades, we considered that their position at the base of the tubulin tree may be the result of long-branch attraction. We therefore reconstructed trees after removing these groups to evaluate whether AtubABC and/or Odin tubulins would then cluster at the base. These trees indicated Odin tubulins as the first group to diverge, followed by AtubC, regardless of whether the outgroup was defined by CetZ, artubulin, or both (Supplementary Figures S8 – S10).

Altogether, we were unable to determine, with very high confidence, a specific evolutionary trajectory for Asgard archaeal and eukaryotic tubulins. While histories involving horizontal transfers are consistent with these results, given the multiple branching patterns of Asgard archaeal tubulins in the tubulin tree and following the long series of recent studies finding an Asgard archaeal ancestry of eukaryotic proteins, we favour a scenario in which duplications and losses were common for tubulin homologues in Asgard archaea, a subset of which was inherited by the eukaryotic lineage, as is discussed in the main text.
